# Supplementary material for: Is obesity more likely among children sharing a household with an older child with obesity? Cross-sectional study of linked National Child Measurement Programme data and electronic health records
Source: BMJ Paediatr Open. 2024 Apr 10;8(1):e002533. doi: 10.1136/bmjpo-2024-002533 (PMC11015308; doi:10.1136/bmjpo-2024-002533)
Supplement: Supplementary data [file bmjpo-2024-002533supp001.pdf]

Is obesity more likely among children sharing a household with an older child with obesity? Cross-sectional study of linked National Child Measurement Programme data and electronic health records

Nicola Firman,[1] Marta Wilk,[1] Milena Marszalek,[1] Lucy Griffiths,[2] Gill Harper,[1] Carol Dezateux[1]

[1] Centre for Primary Care, Wolfson Institute of Population Health, Faculty of Medicine and Dentistry, Queen Mary University of London, Yvonne Carter Building, 58 Turner Street, London, E1 2AB

[2] Swansea University Medical School, Faculty of Medicine, Health & Life Sciences

Supplementary material

Figure S1 – Relationship between address and General Practice (GP) registration dates and Residential Anonymised Linkage Fields (RALFs) at the time of National Child Measurement Programme (NCMP)

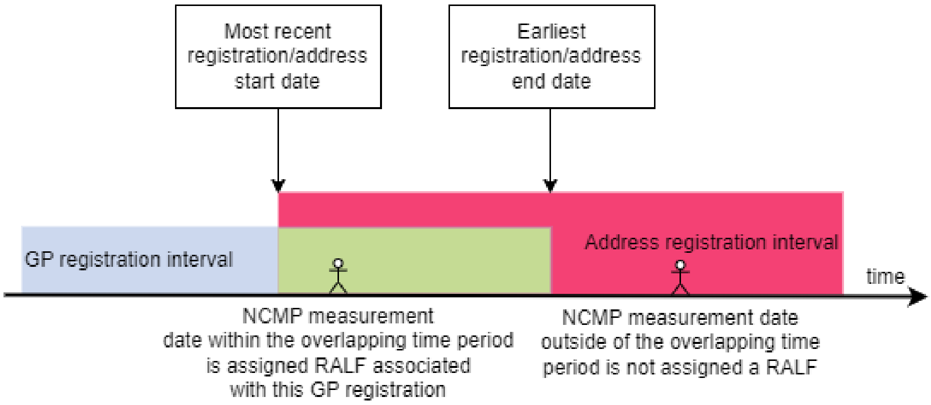

Figure S2 – Identifying National Child Measurement Programme (NCMP) participants' valid General Practice (GP)-recorded Residential Anonymised Linkage Fields (RALFs)

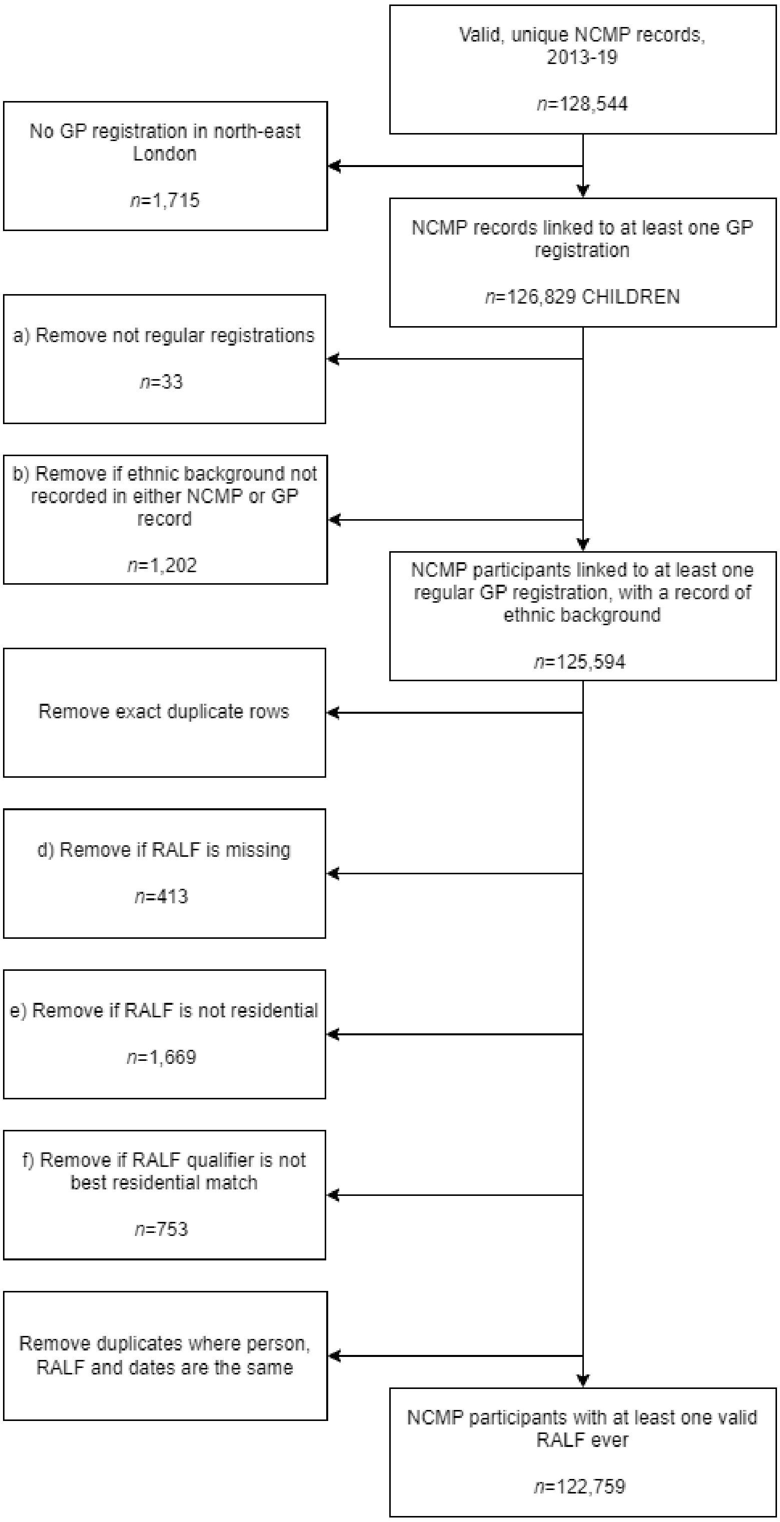

Figure S3 – Identifying National Child Measurement Programme (NCMP) participants sharing a household using Residential Anonymised Linkage Fields (RALFs)

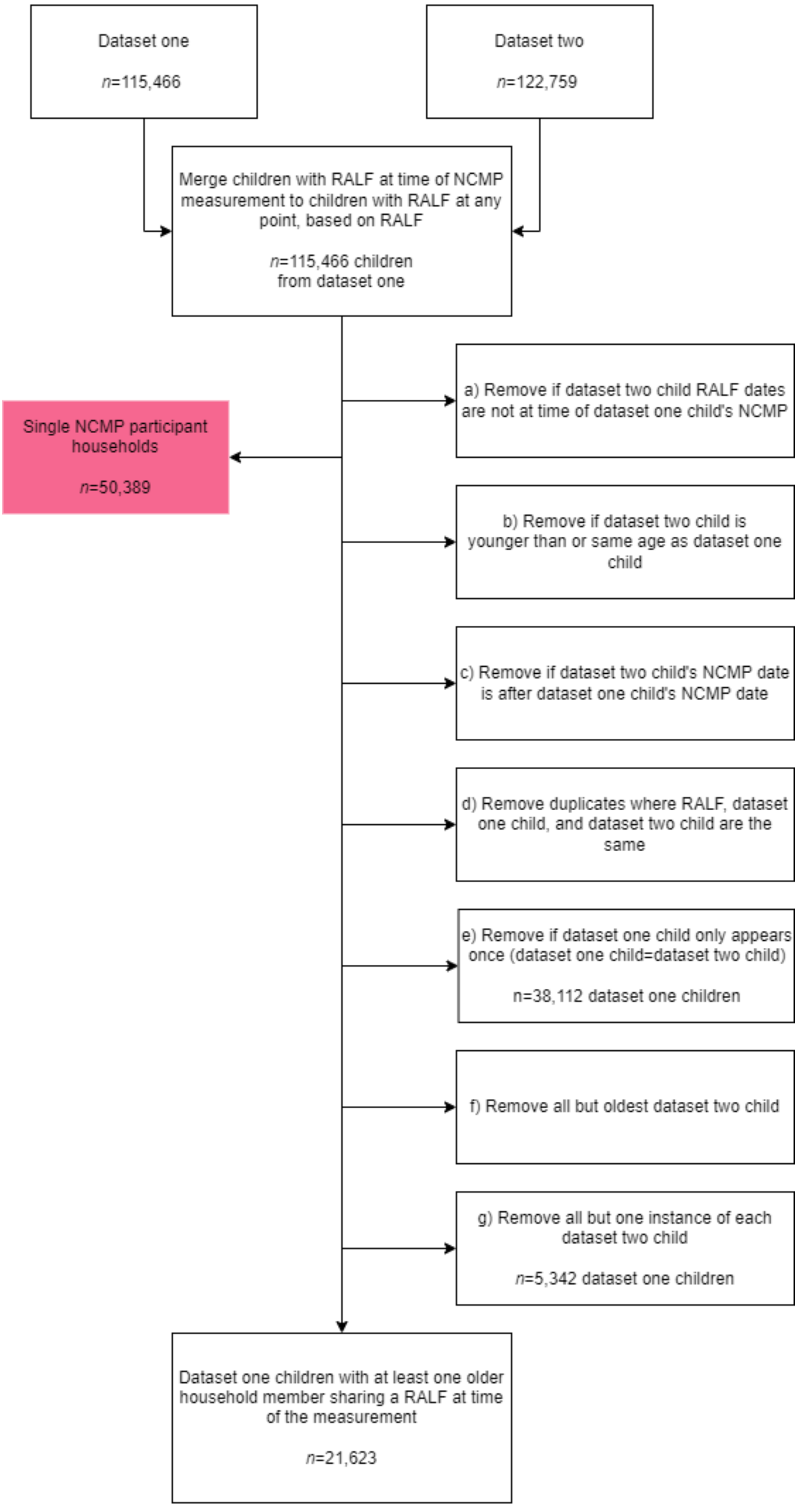

Index children were joined with children in dataset two using Stata's many to many `joinby` function, combining all pairwise combinations on common RALFs. Where an older child matched to multiple index children, only one random pair was retained (n=5,342).

Figure S4 – Identifying all other household members at the time of the index child's NCMP measurement

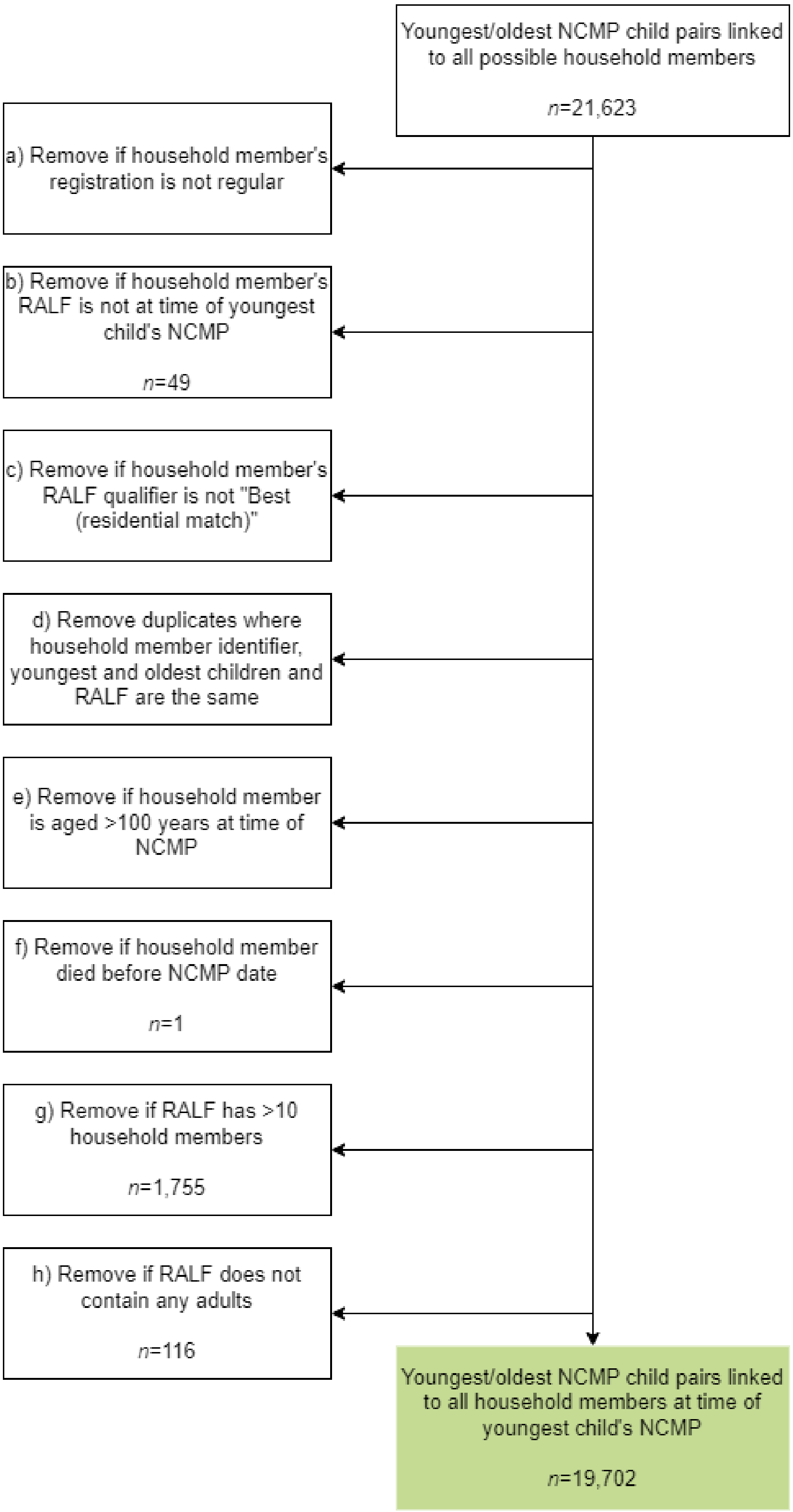

For each household member, age at the time of the youngest child's NCMP measurement was calculated.

Table S1 – Sample characteristics of oldest National Child Measurement Programme participants in the household

The distribution by sex, ethnic background and weight status was broadly representative of NCMP participants in the four local authorities as a whole.

	All (*n*=19,702)			Reception (*n*=6,506)			Year 6 (*n*=13,196)		
	n	%	95% CI[1]	n	%	95% CI[1]	n	%	95% CI[1]
**Sex**									
Male	9975	50.6	49.9,51.3	3268	50.1	48.9,51.3	6707	50.9	50.0,51.7
Female	9727	49.4	48.7,50.1	3238	49.9	48.7,51.1	6489	49.1	48.3,50.0
**School year[2]**									
Reception	6506	33.0	32.3,33.6	6506	100.0				
Year 6	13196	67.0	66.4,67.7				13196	100.0	
**Academic year[3]**									
2013/14	3387	17.2	16.7,17.7	1418	21.8	20.8,22.9	1969	14.9	14.3,15.5
2014/15	3820	19.4	18.9,20.0	1412	21.7	20.7,22.7	2408	18.3	17.6,19.0
2015/16	5888	29.9	29.3,30.5	2075	31.9	30.8,33.0	3813	28.9	28.1,29.7
2016/17	1435	21.0	20.4,21.6	1251	19.2	18.3,20.2	2884	21.9	21.2,22.6
2017/18 & 2018/19	2472	12.5	12.1,13.0	350	5.3	4.9,6.0	2122	16.0	15.5,16.7
**Local authority[4]**									
City & Hackney	5041	25.6	25.0,26.2	1848	28.4	27.4,29.5	3193	24.2	23.5,24.9
Newham	3460	17.5	17.0,18.1	864	13.3	12.5,14.1	2596	19.6	19.0,20.3
Tower Hamlets	6458	32.8	32.1,33.5	2006	30.8	29.6,31.9	4452	33.8	33.0,34.6
Waltham Forest	4743	24.1	23.5,24.7	1788	27.5	26.5,28.6	2955	22.4	21.7,23.1
**Ethnic background[5]**									
White	4503	22.9	22.3,23.4	1873	28.8	27.7,29.9	2630	19.9	19.3,20.6
Mixed and Other	4118	20.9	20.3,21.5	1361	20.9	20.0,21.9	2757	20.9	20.2,21.6
South Asian	6857	34.8	34.1,35.5	1917	29.5	28.4,30.6	4940	37.4	36.6,38.3
Black	4224	21.4	20.9,22.0	1355	20.8	19.8,21.8	2869	21.7	21.1,22.5
**Weight status[6]**									
Underweight	280	1.4	1.3,1.6	97	1.5	1.2,1.8	183	1.4	1.2,1.6
Healthy weight	13938	70.7	70.1,71.4	5299	81.4	80.5,82.4	8639	65.5	64.7,66.3
Overweight	2930	14.9	14.4,15.4	679	10.4	9.7,11.2	2251	17.0	16.4,17.7
Obese	2554	13.0	12.5,13.4	431	6.7	6.1,7.3	2123	16.1	15.5,16.7

[1] Confidence interval. [2] School year of participation in the National Child Measurement Programme (NCMP), Reception participants are aged 4-5 years and Year 6 participants are aged 10-11 years. [3] Academic year of participation in the NCMP. Academic years run from September to July. The 2017/18 and 2018/19 academic years were combined to minimise risk of disclosure of individuals. [4] Local authority of school where child participated in the NCMP. [5] As recorded in the NCMP and, where missing, supplemented with ethnic background as recorded in the child's primary care electronic health record. [6] NCMP-recorded body mass index (BMI) after application of ethnic-specific BMI adjustments, categorised according to UK1990 clinical reference standard: "underweight" (BMI<2nd centile), "healthy weight" (≥2nd to <91st centile), "overweight" (≥91st to <98th centile), or "obese" (≥98th centile).

Table S2 – Weight status distribution of index children participating in the National Child Measurement Programme, using UK1990 clinical and International Obesity Task Force cut-offs

	**All (*n*=19,702)**			**Reception (*n*=13,699)**			**Year 6 (*n*=6,003)**		
	**n**	**%**	**95% CI[1]**	**n**	**%**	**95% CI[1]**	**n**	**%**	**95% CI[1]**
**UK1990 clinical reference standard**									
Underweight	270	1.4	1.2,1.5	194	1.4	1.2,1.6	76	1.3	1.0,1.6
Healthy weight	15005	76.2	75.6,76.8	11025	80.5	79.9,81.2	3980	66.3	65.1,67.5
Overweight	2372	12.0	11.5,12.4	1399	10.2	9.7,10.7	973	16.1	15.2,17.0
Obese	2055	10.4	10.0,10.9	1081	7.9	7.4,8.3	974	16.3	15.4,17.3
**International Obesity Task Force cut-offs**									
Underweight	1409	7.1	6.8,7.5	1037	7.6	7.1,8.0	372	6.2	5.6,6.8
Healthy weight	13339	67.7	67.0,68.4	9682	70.7	69.9,71.4	3657	60.9	59.7,62.1
Overweight	3485	17.7	17.2,18.2	2054	15.0	14.4,15.6	1431	23.8	22.8,24.9
Obese	1469	7.5	7.1,7.8	926	6.7	6.4,7.2	543	9.1	8.3,9.8

[1] Confidence interval.

Table S3 – Household characteristics of children living in households with two National Child Measurement Programme participants, by index child's weight status[1]

	**Youngest child (*n*=19 702)**								
	**Underweight & Healthy weight (*n*=15 275)**			**Overweight (*n*=2 372)**			**Obese (*n*=2 055)**		
**Number of children in the household**									
2	4 992	32.7	31.9,33.4	789	33.3	31.6,35.4	668	32.5	30.5,34.5
3	5 596	36.6	35.8,37.4	899	37.9	35.9,39.8	733	35.7	33.7,37.8
4 or more	4 687	30.7	30.0,31.5	684	28.8	26.9,30.6	654	31.8	29.8,33.8
**Household composition**									
Adults with children	11 593	75.9	75.2,76.6	1 797	75.8	74.0,77.5	1 586	77.2	75.3,78.9
Single adult with children	2 282	14.9	14.4,15.5	333	14.0	12.7,15.5	258	12.5	11.2,14.0
Three generation and skipped generation	1 400	9.2	8.7,9.6	242	10.2	9.0,11.5	211	10.3	9.1,11.7
**Property classification**									
Flat	7 887	51.6	50.8,52.4	1 253	52.8	50.8,54.8	1 120	54.5	52.3,56.6
Terraced house	6 375	41.7	41.0,42.5	970	40.9	38.9,42.9	809	39.4	37.3,41.5
Other	1 013	6.6	6.2,7.0	149	6.3	5.4,7.3	126	6.1	5.2,7.3

[1] National Child Measurement Programme (NCMP)-recorded body mass index (BMI) after application of ethnic-specific BMI adjustments, categorised according to UK1990 clinical reference standard: "underweight or healthy weight" (<91st centile), "overweight" (≥91st to <98th centile), or "obese" (≥98th centile). [2] Confidence interval.

Table S4 – Characteristics of index and oldest child pairs, by index child's weight status[1]

	Underweight & Healthy weight (*n*=15 275)			Overweight (*n*=2 372)			Obese (*n*=2 055)		
	**n**	**%**	**95% CI[2]**	**n**	**%**	**95% CI[2]**	**n**	**%**	**95% CI[2]**
**Oldest child's weight status[1]**									
Underweight	256	1.7	1.5,1.9	13	0.5	0.3,0.9	11	0.5	0.3,1.0
Healthy weight	11 652	76.3	75.6,76.9	1 371	57.8	55.7,59.7	915	44.5	42.5,46.8
Overweight	1 955	12.8	12.3,13.3	528	22.3	20.8,24.1	447	21.8	19.9,23.5
Obese	1 412	9.2	8.8,9.7	460	19.4	17.8,21.0	682	33.2	31.2,35.2
**Sex concordance[3]**									
Different	7 681	50.0	49.4,51.0	1 174	49.5	47.5,51.5	1 049	51.0	48.8,53.1
Same	7 594	49.7	49.0,50.6	1 198	50.5	48.5,52.5	1 006	49.0	46.9,51.2
**Time difference (months)[4]**									
Median (IQR[5])	23.3	12.6,33.1		23.4	12.6,33.0		22.9	12.5,31.9	

[1] National Child Measurement Programme (NCMP)-recorded body mass index (BMI) after application of ethnic-specific BMI adjustments, categorised according to UK1990 clinical reference standard: "underweight" (BMI<2nd centile), "healthy weight" (≥2nd to <91st centile), "overweight" (≥91st to <98th centile), or "obese" (≥98th centile). [2] Confidence interval. [3] Whether the youngest and oldest child were of the same or different sex. [4] Time difference, calculated in months, between the oldest and youngest child's NCMP measurements. [5] Interquartile range.

Table S5 – Univariable and multivariable odds ratios estimating the likelihood of obesity[1] among index children living in households with two National Child Measurement Programme participants

	**All (*n*=19 702)**				**Reception (*n*=13 699)**				**Year 6 (*n*=6 003)**			
	**Univariable**		**Multivariable[2]**		**Univariable**		**Multivariable[3]**		**Univariable**		**Multivariable**	
	**OR[5]**	**95% CI[6]**	**OR[5]**	**95% CI[6]**	**OR[5]**	**95% CI[6]**	**OR[5]**	**95% CI[6]**	**OR[5]**	**95% CI[6]**	**OR[5]**	**95% CI[6]**
**Oldest child's weight status[7]**												
Underweight & Healthy weight (reference)	1		1		1		1		1		1	
Overweight	2.58	2.29,2.92	2.33	2.06,2.64	2.46	2.09,2.90	2.20	1.87,2.60	2.57	2.14,3.08	2.46	2.05,2.96
Obese	5.23	4.68,5.84	4.59	4.10,5.14	5.00	4.31,5.80	4.41	3.79,5.13	5.05	4.27,5.97	4.82	4.07,5.71
**Number of children in the household**												
2 (reference)	1		1		1		1		1			
3	0.98	0.87,1.09	0.87	0.77,0.98	1.00	0.86,1.17	0.83	0.70,0.97	1.03	0.87,1.21		
4 or more	1.05	0.94,1.18	0.96	0.84,1.08	1.13	0.97,1.32	0.91	0.78,1.08	1.14	0.96,1.35		
**Household composition**												
Adults with children (reference)	1				1				1			
Single adult with children	0.83	0.73,0.96			0.75	0.61,0.91			0.91	0.74,1.40		
Three and skipped generation	1.08	0.93,1.26			1.04	0.85,1.29			1.12	0.89,1.40		
**Sex**												
Male (reference)	1		1		1		1		1		1	
Female	0.74	0.68,0.81	0.73	0.67,0.81	0.81	0.71,0.92	0.80	0.71,0.91	0.66	0.58,0.76	0.65	0.56,0.75
**Ethnic background[8]**												
White (reference)	1		1		1		1		1		1	
Mixed and Other	1.25	1.07,1.45	1.17	1.00,1.37	1.13	0.91,1.40	1.10	0.88,1.36	1.36	1.08,1.70	1.25	0.99,1.57
South Asian	2.20	1.94,2.49	1.89	1.64,2.19	2.40	2.02,2.84	2.09	1.75,2.50	2.02	1.67,2.46	1.67	1.37,2.04
Black	0.84	0.71,0.98	0.78	0.66,0.93	0.61	0.48,0.78	0.63	0.49,0.81	1.04	0.83,1.31	0.96	0.76,1.21
**School year[9]**												
Reception (reference)	1		1									
Year 6	2.26	2.06,2.48	2.21	2.00,2.43								

	**All (*n*=19 702)**				**Reception (*n*=13 699)**				**Year 6 (*n*=6 003)**			
	**Univariable**		**Multivariable[2]**		**Univariable**		**Multivariable[3]**		**Univariable**		**Multivariable**	
	**OR[5]**	**95% CI[6]**	**OR[5]**	**95% CI[6]**	**OR[5]**	**95% CI[6]**	**OR[5]**	**95% CI[6]**	**OR[5]**	**95% CI[6]**	**OR[5]**	**95% CI[6]**
**Academic year[10]**												
2013/14	0.70	0.32,1.52			0.86	0.37,1.99						
2014/15	0.83	0.60,1.16			1.01	0.69,1.49			1.17	0.58,2.34		
2015/16	0.94	0.80,1.11			1.16	0.95,1.43			0.93	0.70,1.24		
2016/17	0.91	0.80,1.03			1.01	0.85,1.21			0.94	0.78,1.15		
2017/18	0.90	0.81,1.01			0.93	0.79,1.08			0.94	0.80,1.11		
2018/19 (reference)	1				1				1			
**Local authority[11]**												
City & Hackney	0.74	0.66,0.84	1.00	0.87,1.14	0.67	0.57,0.80			0.84	0.70,1.01		
Newham (reference.)	1		1		1				1			
Tower Hamlets	1.15	1.02,1.30	0.96	0.84,1.10	1.25	1.06,1.47			1.13	0.92,1.38		
Waltham Forest	0.74	0.65,0.84	0.87	0.76,0.99	0.69	0.58,0.83			0.76	0.63,0.92		
**Sex concordance[12]**												
Different (reference.)	1				1				1			
Same	0.97	0.88,1.06			0.96	0.85,1.08			1.00	0.87,1.14		
**Time difference (months)[13]**												
	1.00	0.99,1.00			0.99	0.99,1.00			1.00	0.99,1.00		

[1] National Child Measurement Programme (NCMP)-recorded body mass index (BMI) after application of ethnic-specific BMI adjustments, categorised according to UK1990 clinical reference standard: "obese" (≥98th centile). [2] Mutually adjusting for oldest child's weight status, number of children in the household, sex, ethnic background and school year. [3] Mutually adjusting for the oldest child's weight status, number of children in the household, sex and ethnic background. [4] Mutually adjusting for the oldest child's weight status, sex and ethnic background. [5] Odds ratio. [6] Confidence interval. [7] National Child Measurement Programme (NCMP)-recorded body mass index (BMI) after application of ethnic-specific BMI adjustments, categorised according to UK1990 clinical reference standard: "underweight or healthy weight" (<91st centile), "overweight" (≥91st to <98th centile), or "obese" (≥98th centile). [8] As recorded in the NCMP and, where missing, supplemented with ethnic background as recorded in the child's primary care electronic health record. [9] School year of participation in the National Child Measurement Programme (NCMP), Reception participants are aged 4-5 years and Year 6 participants are aged 10-11 years. [10] Academic year of participation in the National Child Measurement Programme (NCMP). Academic years run from September to July. [11] Local authority of school where child participated in the NCMP. [12] Whether the youngest and oldest child were of the same or different sex. [13] Time difference, calculated in months, between the oldest and youngest child's NCMP measurements. Cells highlighted in bold indicate occasions where the estimates are significantly difference to the reference group in the multivariable models.
