# Supplementary material for: Is obesity more likely among children sharing a household with an older child with obesity? Cross-sectional study of linked National Child Measurement Programme data and electronic health records
Source: BMJ Paediatr Open. 2024 Apr 10;8(1):e002533. doi: 10.1136/bmjpo-2024-002533 (PMC11015308; doi:10.1136/bmjpo-2024-002533)
Supplement: Author's manuscript [file bmjpo-2024-002533.draft_revisions.pdf]

BMJ Paediatrics Open

BMJ Paediatrics Open is committed to open peer review. As part of this commitment we make the peer review history of every article we publish publicly available.

When an article is published we post the peer reviewers' comments and the authors' responses online. We also post the versions of the paper that were used during peer review. These are the versions that the peer review comments apply to.

The versions of the paper that follow are the versions that were submitted during the peer review process. They are not the versions of record or the final published versions. They should not be cited or distributed as the published version of this manuscript.

BMJ Paediatrics Open is an open access journal and the full, final, typeset and author-corrected version of record of the manuscript is available on our site with no access controls, subscription charges or pay-per-view fees (http://bmjpaedsopen.bmj.com).

If you have any questions on BMJ Paediatrics Open's open peer review process please email info.bmjpo@bmj.com

BMJ Paediatrics Open

Is obesity more likely among children sharing a household with an older child with obesity? Cross-sectional study of linked National Child Measurement Programme data and electronic health records

Journal:	*BMJ Paediatrics Open*
Manuscript ID	bmjpo-2024-002533
Article Type:	Original research
Date Submitted by the Author:	24-Jan-2024
Complete List of Authors:	Firman, Nicola; Queen Mary University of London, Wolfson Institute of Population Health<br>Marszalek, Milena; Queen Mary University of London, Wolfson Institute of Population Health<br>Wilk, Marta; Queen Mary University of London, Wolfson Institute of Population Health<br>Harper, Gill; Queen Mary University of London, Wolfson Institute of Population Health<br>Dezateux, Carol; Queen Mary University of London, Wolfson Institute of Population Health<br>Griffiths, Lucy; Swansea University, Faculty of Medicine, Health & Life Sciences
Keywords:	Obesity, Epidemiology

SCHOLARONE™
Manuscripts

I, the Submitting Author has the right to grant and does grant on behalf of all authors of the Work (as defined in the below author licence), an exclusive licence and/or a non-exclusive licence for contributions from authors who are: i) UK Crown employees; ii) where BMJ has agreed a CC-BY licence shall apply, and/or iii) in accordance with the terms applicable for US Federal Government officers or employees acting as part of their official duties; on a worldwide, perpetual, irrevocable, royalty-free basis to BMJ Publishing Group Ltd ("BMJ") its licensees and where the relevant Journal is co-owned by BMJ to the co-owners of the Journal, to publish the Work in this journal and any other BMJ products and to exploit all rights, as set out in our licence.

The Submitting Author accepts and understands that any supply made under these terms is made by BMJ to the Submitting Author unless you are acting as an employee on behalf of your employer or a postgraduate student of an affiliated institution which is paying any applicable article publishing charge ("APC") for Open Access articles. Where the Submitting Author wishes to make the Work available on an Open Access basis (and intends to pay the relevant APC), the terms of reuse of such Open Access shall be governed by a Creative Commons licence – details of these licences and which Creative Commons licence will apply to this Work are set out in our licence referred to above.

Other than as permitted in any relevant BMJ Author's Self Archiving Policies, I confirm this Work has not been accepted for publication elsewhere, is not being considered for publication elsewhere and does not duplicate material already published. I confirm all authors consent to publication of this Work and authorise the granting of this licence.

Confidential: For Review Only

Is obesity more likely among children sharing a household with an older child with obesity? Cross-sectional study of linked National Child Measurement Programme data and electronic health records

Nicola Firman (ORCiD 0000-0001-5213-5044),[1] Marta Wilk,[1] Milena Marszalek,[1] Lucy Griffiths,[2] Gill Harper,[1] Carol Dezateux[1]

[1] Centre for Primary Care, Wolfson Institute of Population Health, Faculty of Medicine and Dentistry, Queen Mary University of London, Yvonne Carter Building, 58 Turner Street, London, E1 2AB

[2] Swansea University Medical School, Faculty of Medicine, Health & Life Sciences

Corresponding author: Nicola Firman; Centre for Primary Care, Wolfson Institute of Population Health, Faculty of Medicine and Dentistry, Queen Mary University of London, Yvonne Carter Building, 58 Turner Street, London, E1 2AB; Nicola.firman@qmul.ac.uk; 0207 882 6806

The authors declare no competing financial interests.

Confidential: For Review Only

Abstract

Background/objectives

We identified household members from electronic health records linked to National Child Measurement Programme (NCMP) data to estimate the likelihood of obesity among children living with an older child with obesity.

Methods

We included 126,829 NCMP participants in four London boroughs and assigned households from encrypted Unique Property Reference Numbers for 115,466 (91.0%). We categorised the ethnic-adjusted body mass index of the youngest and oldest household children (underweight/healthy weight<91st, ≥91st overweight<98th, obesity≥98th centile) and estimated adjusted odds ratios (OR) and 95% confidence intervals (CI) of obesity in the youngest child by the oldest child's weight status, adjusting for number of household children (two, three or ≥4), youngest child's sex, ethnicity, and school year of NCMP participation.

Results

We identified 19,702 households shared by two or more NCMP participants (youngest children: 51.2% male). One third of youngest children with obesity shared a household with another child with obesity (33.2%; 95% CI: 31.2,35.2), compared with 9.2% (8.8,9.7) of youngest children with a healthy weight. Youngest children living with an older child with a BMI considered overweight (OR: 2.33; 95% CI: 2.06,2.64) or obese (4.59, 4.10,5.14) were more likely to be living with obesity.

Conclusions

Identifying children sharing households in primary care and linking to the NCMP provides novel insights into the shared weight status of children in the same household. Qualitative research is needed to understand how food practices vary by household characteristics to improve our understanding of how the home environment influences childhood obesity.

Key words

Childhood, obesity, households, BMI, electronic health records, record linkage

Key messages

There is a evidence to suggest that children living with older siblings with obesity are more likely to be living with obesity themselves. Less is known about the associations between the obesity status of child household members who are not biologically related.

What this study adds

We examined associations between child household weight status using novel linkages between school measurement and electronic health records. We showed that younger children living with an older child with obesity were more than four times more likely to live with obesity than those living with an older child with a healthy weight.

How this study might affect research, practice or policy

A household-level approach may potentially reach more children living with, and at risk of, obesity, to encompass a broader range of factors which may or may not influence obesity among all resident children.

Confidential: For Review Only

Introduction

Childhood obesity is a major public health concern globally and reflects a complex number of factors, in particular socio-economic inequalities.(1) In England, more than one quarter of children leave primary school with overweight or obesity at a level of severity defined as in need of clinical intervention.(2)

A child's health, including their weight status, is significantly affected by the environment in which they live. Better understanding of households, their composition, and the health of children and adults who share households may provide novel actionable insights to address unhealthy weight in childhood.

Research has shown that child obesity is associated with parental obesity, where parental overweight or obesity is associated with an increased risk of obesity in their child.(3) This relationship is stronger for mothers than fathers.(4, 5) Less is known about the associations between the obesity status of child household members. Research investigating associations between siblings' weight status has reported inconsistent results.(6-13)

A 2023 systematic review of 13 studies identified that siblings' health-related behaviours and weight-related outcomes varied according to sibling sex and birth order.(14) Our understanding of how household composition, including presence of a sibling or other household children, as well as their weight status, influences childhood obesity could be improved with further research which includes all child household members and not just those who are biologically-related.

We identified individuals sharing a household using electronic health records and linked this to school measurement programme data to estimate the likelihood of obesity among children living with an older child with obesity. We also investigated whether household composition and size, and dwelling type, influenced the likelihood of childhood obesity.

Methods

Study population

Children in the first (Reception year) and last (Year 6) years of primary school are invited to participate in the National Child Measurement Programme (NCMP), which measures the height and weight of 4-5- and 10-11-

Confidential: For Review Only

year-olds attending state-maintained schools in England. We linked 126,829 of 128,544 (98.7%) NCMP records from four north-east London (NEL) local authorities (City & Hackney, Newham, Tower Hamlets, and Waltham Forest) to general practice (GP) electronic health records (EHRs) via the Discovery Data Service (DDS).(15)

Data sources

We obtained pseudonymised NCMP data for the 2013/14-2018/19 academic years under data processing agreements with each local authority public health department. As the available date of NCMP measurement was restricted to month and year, we randomly assigned a day of measurement within term-time, excluding weekends and bank holidays to avoid a spurious reduction in variance in age at measurement occasioned by using the same fixed date of measurement for every child (R Studio; version 1.0.153; code available on request).

Pseudonymised data were provided from the DDS which receives primary care EHR data on a daily basis from all GPs in NEL. Demographic and clinical data recorded up to 1st November 2021 were extracted for NCMP participants successfully linked to the DDS via pseudonymised NHS numbers created using OpenPseudonymiser software.(16) All data were extracted and managed according to UK National Health Service (NHS) information governance requirements.(17)

Data processing

Residential Anonymised Linkage Fields

Every addressable location in Great Britain is assigned a Unique Property Reference Number (UPRN). UPRNs identify a place of residence at a granular level, identifying individual properties, for example houses or flats within a block or building shell. UPRNs are allocated to GP-recorded addresses using the validated ASSIGN algorithm,(18) and pseudonymised into Residential Anonymised Linkage Fields (RALFs) within DDS, using a study-specific encryption key.

Identifying household members at the child's school measurement date

A household can only be defined at a single point in time because people living at an address may change over time whilst the UPRN assigned to the residential dwelling stays the same.

Confidential: For Review Only

A data extract containing all RALFs associated with any address(es) recorded in a child's EHR was extracted. The file contained start and end dates of patient registration (enrolment) with the GP as well as address start and end dates. Address start and end dates refer to the dates at which a patient lived at a particular address. In most cases, these align with GP registration dates, but could differ, if for example, a patient moved house but remained registered with the same GP.

Figure 1 describes the process for deciding which, if any, of the child's RALFs was the place of residence at the time of their NCMP measurement. If the NCMP date of measurement took place between the most recent of the registration and address start dates and the earliest of the registration and address end dates, the RALF was considered to be the place of residence at the time of NCMP (supplementary Figure S1). Children without a RALF at the time of their NCMP were excluded (*n*=5,519). We retained 115,466 children with a RALF at the time of their NCMP measurement (referred to hereafter as index children).

Identifying other National Child Measurement Programme participants in the household

Similar steps were taken to identify other NCMP participants sharing a household with index children. We started with 122,759 NCMP participants with at least one RALF (supplementary Figure S2) and included in "dataset two".

Child pairs were excluded if the index child's NCMP measurement date did not fall within dataset two child's RALF dates (supplementary Figure S3). Pairs were also excluded if the dataset two child was younger than the index child or if the dataset two child's NCMP measurement was after the index child's. This process found the youngest index child and identified the oldest NCMP participant living in the same household at the time of the index child's NCMP measurement. Of 128,554 NCMP participants, 21,623 youngest/oldest child pairs were identified.

Identifying other household members and deriving household characteristics

We identified all people who had ever lived at any of the RALFs identified in the dataset of youngest/oldest child pairs. Steps were taken to determine household members at the time of the index child's NCMP measurement (supplementary Figure S4). Full household information was identified for 19,702 youngest/oldest child pairs.

Confidential: For Review Only

Outcome of interest

Obesity status of the index child was defined by the UK1990 clinical reference standard.(19) After application of ethnic-specific BMI adjustments,(20) a binary variable indicating obesity was defined as a BMI greater than or equal to the 98th age- and sex-specific centile. The index child's ethnic-adjusted BMI *z*-score was a secondary outcome.

Explanatory variables

Ethnic-specific BMI adjustments(20) were applied to the older child's BMI, and weight status determined according to the UK1990 clinical reference standard(19) categorising BMI into one of four mutually exclusive groups: "underweight" (<2nd centile), "healthy weight" (≥2nd to <91st centile), "overweight" (≥91st to <98th centile) or "obese" (≥98th centile). The older child's BMI *z*-score was also considered as an explanatory variable.

NCMP-recorded sex, local authority of the school where the child participated in the NCMP, academic year (September to July) and school year (Reception/Year 6) of participation in the NCMP were explanatory variables.

NCMP-recorded ethnic background was grouped into four mutually exclusive groups(21): White ('White British', 'White Irish', or 'any other White background'); Black ('Black African', 'Black Caribbean', or 'any other Black background'); South Asian ('Indian', 'Pakistani', 'Bangladeshi' or 'Sri Lankan'); and a combination of Mixed and Other ('any other ethnic background', 'mixed ethnicity', 'Chinese' or 'Asian other'). Where ethnic background was missing or not stated in the NCMP, ethnic background as recorded in the EHR (*n*=11,077) was used.

An area-level measure of relative deprivation - Index of Multiple Deprivation (IMD) decile(22) - was assigned to each NCMP record based on the postcode of the child's home address. IMD decile was concatenated into five quintiles ranging from most (1) to least deprived (5).

A categorical variable was derived from a count of people assigned the same RALF as the NCMP participant, grouping household size as follows: 2; 3-4; 5-6; 7-10.

https://mc.manuscriptcentral.com/bmjpo

Confidential: For Review Only

We categorising household composition using a modified Harper and Mayhew method(23) by counting the number of household members in three age brackets: 0-17 years (children), 18-64 years (working age adults) and 65 or older (older adults) and grouping into: working-aged adults with children; a single working-age adult with children; at least one working-age and one older adult with children (three generation household), or at least one older adult with children (skipped generation household).

The property classification, as given by the ASSIGN algorithm, categorised properties into three groups: flats, terraced houses, and other.

Sex concordance was coded either the same (when both children shared the same sex) or different (when the two children had differing sexes). The time difference between the youngest and oldest children's NCMP measurements was calculated as the time in months between the two measurements.

Statistical analyses

We estimated the prevalence of obesity among children living with an older NCMP participant and explored variation by the weight status of the older child. We used binary logistic regression to estimate the likelihood of obesity in the index child (odds ratio [OR] and 95% confidence interval [CI]) by the older child's weight status, after accounting for individual and household characteristics. We conducted linear regression to estimate the effect of a one unit increase in the oldest child's BMI *z*-score on the index child's BMI *z*-score. All analyses, conducted using Stata (MP/15.0), were stratified by school year.

Patient and public involvement

This research was done without patient or public involvement. Neither were invited to comment on the study design and were not consulted to develop relevant outcomes or interpret results.

Ethics approval

The analyses of linked pseudonymised NCMP and GP data were approved by the respective data controllers under data processing agreements which allow linkage of pseudonymised NCMP data between the research organisation and each local authority public health team. This study is a secondary analysis of de-identifiable data and no further ethics approval was required.

https://mc.manuscriptcentral.com/bmjpo

Confidential: For Review Only

Results

Index children were, by study design, more likely to take part in the NCMP in the Reception school year and in the more recent academic years (Table 1). Similarly, the oldest children were more likely to take part in the NCMP in Year 6 and in the earlier academic years (supplementary Table S1). Three quarters lived in households with adults of working age only, and more than half lived in flats (Table 2).

A greater proportion of index children with obesity were male, participating in the NCMP in Year 6 and in Tower Hamlets and Newham, and from South Asian ethnic backgrounds, compared with index children with underweight or a healthy weight (Table 3).

There was no variation in the number of children sharing a household or property classification by weight status (Table S2). A lower proportion of index children living with obesity lived in households with a single adult (12.5%; 95% CI: 11.2,14.0) compared with the proportion among children with an underweight/healthy weight status (14.9%; 14.4,15.5).

One fifth and one third of index children living with obesity shared a household with another child with overweight or obesity, respectively, higher than those with underweight or of a healthy weight (Table S3; Figure 2). Sex concordance, nor time difference between the index and older children's NCMP measurement dates, did not vary by weight status of the index child.

in adjusted analyses, index children living with an older child with overweight or obesity were more likely to be living with obesity. Conversely, those sharing a household with two other children were less likely to be living with obesity (Figure 3; uni- and multi-variable results are presented in Table S4).

In multivariable linear regression, a one unit increase in the oldest child's BMI *z*-score was associated with a 0.32 (95% CI: 0.30,0.33) increase in the index child's BMI *z*-score. Similarly, in linear regression models stratified by the school year of participation in the NCMP, a one unit increase in the oldest child's BMI *z*-score predicted a 0.28 (0.27,0.29) and 0.38 (0.35,0.40) increase in Reception and Year 6 index child's BMI *z*-scores, respectively.

Confidential: For Review Only

Discussion

Summary of key findings

We examined associations between child household weight status using novel linkages between school measurement and EHRs. We showed that younger children living with an older child with obesity were more than four times more likely to live with obesity than those living with an older child with a healthy weight.

Strengths and limitations

We used ethnic-specific BMI adjustments and the UK1990 clinical thresholds to identify children with obesity considered in need of clinical intervention in an ethnically-diverse area of London with high levels of childhood obesity.

We used linked NCMP records of weight status as we have previously shown that GP EHRs do not contain accurate, up-to-date child measurement data and are biased to children at both extremes of the BMI distribution.(24) This resulted in exclusion of 30,552 NCMP participants who did not live with another NCMP participant in the 2013-2019 academic years.

We used a robust methodology to identify household members at the time of the NCMP measurement. The ASSIGN algorithm has been shown to match 98.6% of primary care patient addresses to UPRNs.(18) We adopted a conservative approach to identify “true” household members, by excluding NCMP participants living in large or non-residential households. It is possible that we included patients who no longer live at their registered address (so would not consult with their registered GP). There is also likely to be a time lag between a patients’ GP registrations, and a period of time where a patient has moved on from an area but remains registered with a GP. Hence, we may have overestimated the true number of household members.

We were not able to determine whether child household members were biologically related. Similarly, we were not able to identify biological parents and account for parental BMI in our analyses. To our knowledge, this is the first time that other children in the household (as opposed to biologically-related siblings) have been accounted for in analyses of childhood obesity. Children are increasingly living in blended families including household members with no biological relationship. Multi-family households (consisting of two or more families) were the fastest growing household type in the UK between 1999 and 2019.(25)

https://mc.manuscriptcentral.com/bmjpo

Confidential: For Review Only

Comparison with existing literature

Our findings support those reporting an increased likelihood of obesity among children living with other children with obesity.(12, 14) Children in the same household spend their time together and share the same resources, which supports the notion of the 'shared home environment'.(26) Siblings eat similar diets, and participate in similar levels of physical activity and sedentary behaviours.(14) Others note that older children are important influencers in children's health-related behaviours, particularly healthy eating. Younger children want to copy the behaviours of their older siblings, explaining the positive correlation between both children's healthy and unhealthy behaviours.(27, 28)

Implications for research, policy and practice

Our findings highlight the importance of taking a household perspective in tackling childhood obesity. A household-level approach may potentially reach more children living with, and at risk of, obesity, to encompass a broader range of factors which may or may not influence obesity among all resident children.

Routinely collected EHRs provide a limited view of the home environment, and further qualitative research is necessary to fully understand who the decision-makers are, and how practices and attitudes relating to food purchasing and diet, as well as physical activity opportunities, are negotiated on a daily basis.

Conclusion

Children living with an older child with obesity are more likely to be living with obesity. Early intervention should be approached from a household perspective which takes into account the roles of, and implications for, all household members.

https://mc.manuscriptcentral.com/bmjpo

Confidential: For Review Only

Funding

This work was supported by ADR UK (Administrative Data Research UK), an Economic and Social Research Council investment (part of UK Research and Innovation) [Grant number: ES/X00046X/1]. This research was also supported by a grant from Barts Charity ref: MGU0419. This work was supported by Health Data Research UK, which is funded by the UK Medical Research Council, Engineering and Physical Sciences Research Council, Economic and Social Research Council, Department of Health and Social Care (England), Chief Scientist Office of the Scottish Government Health and Social Care Directorates, Health and Social Care Research and Development Division (Welsh Government), Public Health Agency (Northern Ireland), British Heart Foundation and Wellcome. This work was supported by the UK Prevention Research Partnership (MR/S037527/1), which is funded by the British Heart Foundation, Cancer Research UK, Chief Scientist Office of the Scottish Government Health and Social Care Directorates, Engineering and Physical Sciences Research Council, Economic and Social Research Council, Health and Social Care Research and Development Division (Welsh Government), Medical Research Council, National Institute for Health Research, Natural Environment Research Council, Public Health Agency (Northern Ireland), The Health Foundation and Wellcome.

Competing interests

The authors declare no competing financial interests.

Author contributions

CD obtained funding for the study. All authors conceptualised and designed the analyses. NF carried out the literature search, conducted the analyses, generated tables and figures and drafted the initial manuscript. All authors contributed to the development of the methodology, interpretation of analyses and reviewed and revised the manuscript. All authors were involved in writing the paper and had final approval of the submitted and published manuscript. The corresponding author attests that all listed authors meet authorship criteria and that no others meeting the criteria have been omitted.

Data sharing

Access to primary care data is enabled by data sharing agreements between the Discovery Data Service and the data controllers. The Discovery Programme Board has approved data access by the REAL Child Health programme team for research on the condition that it is not onwardly shared. NCMP data were accessed under data processing agreements with each of the local authorities as data controllers in line with Public Health England guidance. These agreements preclude onward sharing of data.

Acknowledgements

The authors are grateful to the local authority public health teams for providing pseudonymised NCMP data, school nurses and NCMP data collection teams, and NCMP participants. We are grateful to Professor Mario Cortina-Borja (University College London Great Ormond Street Institute of Child Health) for supporting the development of an algorithm to randomly assign a day of measurement. We are grateful to the data controllers of the Discovery Programme for allowing access to primary care data and to colleagues in the

https://mc.manuscriptcentral.com/bmjpo

Confidential: For Review Only

Clinical Effectiveness Group for their expert support. This work uses data provided by patients and collected by the NHS as part of their care and support.

Confidential: For Review Only

Tables

Table 1 – Sample characteristics of index children participating in the National Child Measurement Programme

	All (*n*=19,702)			Reception (*n*=13,699)			Year 6 (*n*=6,003)		
	**n**	**%**	**95% CI[1]**	**n**	**%**	**95% CI[1]**	**n**	**%**	**95% CI[1]**
**Sex**									
Male	10079	51.2	50.5,51.9	7005	51.2	50.4,52.1	3074	51.1	49.9,52.4
Female	9623	48.8	48.1,49.5	6694	48.8	47.9,49.6	2929	48.9	47.6,50.1
**School year[2]**									
Reception	13699	69.5	68.9,70.2	13699	100.0				
Year 6	6003	30.5	29.8,31.1				6003	100.0	
**Academic year[3]**									
2031/14 & 2014/15	517	2.6	2.4,2.9	463	3.4	3.1,3.7	54	0.9	0.6,1.2
2015/16	1926	9.8	9.4,10.2	1530	11.2	10.7,11.7	396	6.6	6.0,7.3
2016/17	3751	19.0	18.5,19.6	2748	20.0	19.4,20.7	1003	16.7	15.8,17.7
2017/18	5980	30.4	29.7,31.0	4100	30.0	29.2,30.7	1880	31.3	30.1,32.5
2018/19	7528	38.2	37.5,38.9	4858	35.5	34.7,36.3	2670	44.5	43.2,45.7
**Local authority[4]**									
City & Hackney	4998	25.4	24.8,26.0	3489	25.5	24.7,26.2	1509	25.2	24.1,26.3
Newham	6472	32.9	32.2,33.5	4444	32.5	31.7,33.3	2028	33.8	32.6,35.0
Tower Hamlets	3495	17.7	17.2,18.3	2571	18.7	18.1,19.4	924	15.4	14.5,16.3
Waltham Forest	4737	24.0	23.4,24.6	3195	23.3	22.6,24.0	1542	25.6	24.6,26.8
**Ethnic background[5]**									
White	4615	23.4	22.9,24.0	3240	23.7	22.9,24.4	1375	22.9	21.9,24.0
Mixed and Other	3823	19.4	18.8,19.9	2620	19.1	18.4,19.8	1203	20.0	19.0,21.1
South Asian	6812	34.6	33.9,35.3	4813	35.1	34.3,35.9	1999	33.3	32.1,34.5
Black	4452	22.6	22.0,23.2	3026	22.1	21.4,22.8	1426	23.7	22.7,24.8
**Weight status[6]**									
Underweight	270	1.4	1.2,1.5	194	1.4	1.2,1.6	76	1.3	1.0,1.6
Healthy weight	15005	76.2	75.6,76.8	11025	80.5	79.9,81.2	3980	66.3	65.1,67.5
Overweight	2372	12.0	11.5,12.4	1399	10.2	9.7,10.7	973	16.1	15.2,17.0
Obese	2055	10.4	10.0,10.9	1081	7.9	7.4,8.3	974	16.3	15.4,17.3

[1] Confidence interval. [2] School year of participation in the National Child Measurement Programme (NCMP), Reception participants are aged 4-5 years and Year 6 participants are aged 10-11 years. [3] Academic year of participation in the NCMP. Academic years run from September to July. The 2013/14 and 2014/15 academic years were combined to minimise the risk of disclosing individuals. [4] Local authority of school where child participated in the NCMP. [5] As recorded in the NCMP and, where missing, supplemented with ethnic background as recorded in the child's primary care electronic health record. [6] NCMP-recorded body mass index (BMI) after application of ethnic-specific BMI adjustments, categorised according to UK1990 clinical reference standard: "underweight" (BMI<2nd centile), "healthy weight" (≥2nd to <91st centile), "overweight" (≥91st to <98th centile), or "obese" (≥98th centile).

Confidential: For Review Only

Table 2 – Household characteristics of children living in households with two National Child Measurement Programme participants

	Two NCMP-participant households (*n*=19,702)		
	**n**	**%**	**95% CI[1]**
**IMD quintile[2]**			
1 (most deprived)	10375	52.6	51.9,53.3
2	7836	39.8	39.1,40.5
3	1292	6.6	6.2,6.9
4	156	0.8	0.7,0.9
5 (least deprived)	43	0.2	0.2,0.3
**Number of children in the household**			
2	6449	32.8	32.1,33.4
3	7228	36.6	36.0,37.3
4 or more	6025	30.6	30.0,31.2
**Household composition**			
Working adults with children	14976	76.0	75.4,76.6
Single working age adult with children	2873	14.6	14.1,15.1
Three generation and skipped generation	1853	9.4	9.0,9.8
**Property classification**			
Flat	10260	52.1	51.4,52.8
Terraced house	8154	41.4	40.7,42.1
Other	1288	6.5	6.2,6.9

[1] Confidence interval. [2] 2015 Index of Multiple Deprivation quintile assigned based on the child's home address postcode as recorded by the school where the child participated in the National Child Measurement Programme.

Confidential: For Review Only

Table 3 – Sociodemographic characteristics of index children living in households with two National Child Measurement Programme participants, by index child's weight status[1]

	**Underweight & Healthy weight (*n*=15,275)**			**Overweight (*n*=2,372)**			**Obese (*n*=2,055)**		
	**n**	**%**	**95% CI[2]**	**n**	**%**	**95% CI[2]**	**n**	**%**	**95% CI[2]**
**Sex**									
Male	7636	50.0	49.3,50.8	1256	52.9	50.9,54.9	1187	57.8	55.6,59.8
Female	7639	50.0	49.2,50.7	1116	47.1	45.1,49.1	868	42.2	40.2,44.4
**School year[3]**									
Reception	11219	73.4	72.7,74.1	1399	59.0	57.0,61.0	1081	52.6	50.3,54.6
Year 6	4056	26.6	25.9,27.3	973	41.0	39.0,43.0	974	47.4	45.4,49.7
**Academic year[4]**									
2013/14 & 2014/15	421	2.8	2.5,3.0	49	2.1	1.6,2.7	47	2.2	1.7,3.0
2015/16	1506	9.9	9.4,10.3	219	9.3	8.2,10.5	201	9.8	8.6,11.2
2016/17	2938	19.2	18.6,19.9	434	18.3	16.8,19.9	379	18.3	16.7,20.0
2017/18	4639	30.4	29.7,31.1	741	31.1	29.3,33.0	600	29.2	27.2,31.1
2018/19	5771	37.7	37.0,38.5	929	39.2	37.3,41.2	828	40.5	38.3,42.6
**Local authority[5]**									
City & Hackney	4001	26.2	25.5,26.9	556	23.2	21.5,24.9	441	21.5	19.8,23.3
Newham	4905	32.2	31.4,32.9	822	34.6	32.7,36.5	745	36.3	34.3,38.5
Tower Hamlets	2572	16.8	16.2,17.4	468	19.9	18.4,21.6	455	22.1	20.3,23.9
Waltham Forest	3797	24.8	24.2,25.5	526	22.3	20.7,24.0	414	20.1	18.4,21.9
**Ethnic background[6]**									
White	3739	24.5	23.8,25.2	522	22.0	20.4,23.7	354	17.2	15.6,18.9
Mixed and Other	3052	20.0	19.3,20.6	412	17.4	15.8,18.9	359	17.5	15.9,19.2
South Asian	4677	30.6	29.9,31.3	1082	45.6	43.8,47.8	1053	51.2	49.1,53.4
Black	3807	24.9	24.3,25.6	356	15.0	13.5,16.4	289	14.1	12.6,15.6

[1] National Child Measurement Programme (NCMP)-recorded body mass index (BMI) after application of ethnic-specific BMI adjustments, categorised according to UK1990 clinical reference standard: "underweight or healthy weight" (<91st centile), "overweight" (≥91st to <98th centile), or "obese" (≥98th centile). [2] Confidence interval. [3] School year of participation in the NCMP, Reception participants are aged 4-5 years and Year 6 participants are aged 10-11 years. [4] Academic year of participation in the National Child Measurement Programme (NCMP). Academic years run from September to July. The 2013/14 and 2014/15 academic years were combined to minimise the risk of disclosing individuals. [5] Local authority of school where child participated in the NCMP. [6] As recorded in the NCMP and, where missing, supplemented with ethnic background as recorded in the child's primary care electronic health record.

Confidential: For Review Only

Figure titles and footnotes

Figure 1 – Identifying a valid Residential Anonymised Linkage Field (RALF) at the time of National Child Measurement Programme (NCMP) measurement

Children living in non-residential dwellings or where the UPRN match qualifier was not a "best" residential match were excluded (n=3,903). The match qualifier indicates how close to the place of residence the assigned UPRN is.

Figure 2 – Oldest child's weight status stratified by index child's weight status[1]

[1] National Child Measurement Programme (NCMP)-recorded body mass index (BMI) after application of ethnic-specific BMI adjustments, categorised according to UK1990 clinical reference standard: "underweight or healthy weight" (<91st centile), "overweight" (≥91st to <98th centile), or "obese" (≥98th centile).

Figure 3 – Multivariable[1] odds ratio estimating the likelihood of obesity[2] among the youngest children living in households with two National Child Measurement Programme participants

[1] The model including all households with two National Child Measurement Programme (NCMP) participants mutually adjusted for oldest child's weight status, number of children in the household, sex, ethnic background as recorded in the NCMP and, where missing, supplemented with ethnic background as recorded in the child's primary care electronic health record, school year of participation in the NCMP (Reception participants are aged 4-5 years and Year 6 participants are aged 10-11 years) and local authority of school where child participated in the NCMP. The model which only included households where the youngest child participated in the NCMP in Reception year mutually adjusted for the oldest child's weight status, number of children in the household, sex and ethnic background. The model which only included households where the youngest child participated in the NCMP in Year 6 mutually adjusted for the oldest child's weight status, sex and ethnic background. [2] NCMP-recorded body mass index (BMI) after application of ethnic-specific BMI adjustments, categorised according to UK1990 clinical reference standard: "obese" (≥98th centile). [3] Odds ratio and confidence interval. Odds ratios are plotted on a logarithmic scale.

Confidential: For Review Only

References

1. World Health Organisation. Childhood overweight and obesity 2019 [Available from: https://www.who.int/dietphysicalactivity/childhood/en/.
2. NHS Digital. National Child Measurement Programme, England 2018/19 School Year 2019 [Available from: https://digital.nhs.uk/data-and-information/publications/statistical/national-child-measurement-programme/2018-19-school-year.
3. Whitaker KL, Jarvis MJ, Beeken RJ, Boniface D, Wardle J. Comparing maternal and paternal intergenerational transmission of obesity risk in a large population-based sample. Am J Clin Nutr. 2010;91(6):1560-7.
4. Classen TJ. Measures of the intergenerational transmission of body mass index between mothers and their children in the United States, 1981-2004. Econ Hum Biol. 2010;8(1):30-43.
5. Johnson PCD, Logue J, McConnachie A, Abu-Rmeileh NME, Hart C, Upton MN, et al. Intergenerational change and familial aggregation of body mass index. European Journal of Epidemiology. 2011;27(1):53-61.
6. Kracht CL, Sisson SB. Sibling influence on children's objectively measured physical activity: a meta-analysis and systematic review. BMJ Open Sport & Exercise Medicine. 2018;4(1):e000405.
7. Meller FO, Loret de Mola C, Assuncao MCF, Schafer AA, Dahly DL, Barros FC. Birth order and number of siblings and their association with overweight and obesity: a systematic review and meta-analysis. Nutr Rev. 2018;76(2):117-24.
8. Meller FO, Assunção MCF, Schäfer AA, De Mola CL, Barros AJD, Dahly DL, et al. The influence of birth order and number of siblings on adolescent body composition: evidence from a Brazilian birth cohort study. British Journal of Nutrition. 2015;114(1):118-25.
9. Park SH, Cormier E. Influence of Siblings on Child Health Behaviors and Obesity: A Systematic Review. Journal of Child and Family Studies. 2018;27(7):2069-81.
10. Chanfreau J, Barclay K, Keenan K, Goisis A. Sibling group size and BMI over the life course: Evidence from four British cohort studies. Advances in Life Course Research. 2022;53.
11. Mosli RH, Kaciroti N, Corwyn RF, Bradley RH, Lumeng JC. Effect of Sibling Birth on BMI Trajectory in the First 6 Years of Life. Pediatrics. 2016;137(4):e20152456.
12. Pachucki MC, Lovenheim MF, Harding M. Within-family obesity associations: evaluation of parent, child, and sibling relationships. Am J Prev Med. 2014;47(4):382-91.
13. Khoury P, Morrison JA, Laskarzewski PM, Glueck CJ. Parent-offspring and sibling body mass index associations during and after sharing of common household environments: The princeton school district family study. Metabolism. 1983;32(1):82-9.
14. Park SH, Cormier E, Grove K, Kelley SJ, Faith MS. Health-related behaviors and weight-related outcomes within sibling pairs: A systematic review. Journal of Pediatric Nursing. 2023;73:e154-e63.
15. Discovery Data Service. Discovery Data Service [Available from: https://www.endeavourhealth.org/DDS.html.
16. Hippisley-Cox J. OpenPseudonymiser: University Of Nottingham; [Available from: https://www.openpseudonymiser.org/.
17. NHS England. About information governance: National Health Service; [Available from: https://www.england.nhs.uk/ig/about/.
18. Harper G, Stables D, Simon P, Ahmed Z, Smith K, Robson J, et al. Evaluation of the ASSIGN open-source deterministic address-matching algorithm for allocating unique property reference numbers to general practitioner-recorded patient addresses. Int J Popul Data Sci. 2021;6(1):1674.
19. Scientific Advisory Committee on Nutrition, Royal College of Paediatrics and Child Health. Consideration of issues around the use of BMI centile thresholds for defining underweight, overweight and obesity in children aged 2-8 years in the UK. 2012.
20. Hudda MT, Nightingale CM, Donin AS, Fewtrell MS, Haroun D, Lum S, et al. Body mass index adjustments to increase the validity of body fatness assessment in UK Black African and South Asian children. Int J Obes. 2017;41(7):1048-55.
21. NHS. Ethnic category code: NHS Digital; [Available from: https://www.datadictionary.nhs.uk/data_elements/ethnic_category.html?hl=ethnic%2Ccategory.
22. Department for Communities and Local Government. The English Indices of Deprivation 2015 - Frequently Asked Questions (FAQs). 2016.

Confidential: For Review Only

23. Harper G, Mayhew L. Using Administrative Data to Count and Classify Households with Local Applications. Applied Spatial Analysis and Policy. 2015;9(4):433-62.
24. Firman N, Robson J, Ahmed Z, Boomla K, Dezateux C. Completeness and representativeness of body mass index in children's electronic general practice records: Linked cross-sectional study in an ethnically-diverse urban population in the United Kingdom. Pediatric Obesity. 2021;16(8):e12772.
25. Office for National Statistics. Families and households in the UK: 2019 [Available from: https://www.ons.gov.uk/peoplepopulationandcommunity/birthsdeathsandmarriages/families/bulletins/familiesandhouseholds/2019#multi-family-households-are-the-fastest-growing-household-type-in-the-uk-but-currently-represent-the-smallest-share-of-households.
26. Faith MS, Pietrobelli A, Heo M, Johnson SL, Keller KL, Heymsfield SB, et al. A twin study of self-regulatory eating in early childhood: estimates of genetic and environmental influence, and measurement considerations. International Journal of Obesity. 2012;36(7):931-7.
27. Osai KV, Dorsch TE, Whiteman SD. “To Be, or Not to Be, That Is the Question”: Modeling and Differentiation Among Siblings Participating in Organized Youth Sport. Journal of Sport & Exercise Psychology. 2020;42(6):500-10.
28. Whiteman SD, Christiansen A. Processes of Sibling Influence in Adolescence: Individual and Family Correlates*. Family Relations. 2008;57(1):24-34.

Confidential: For Review Only

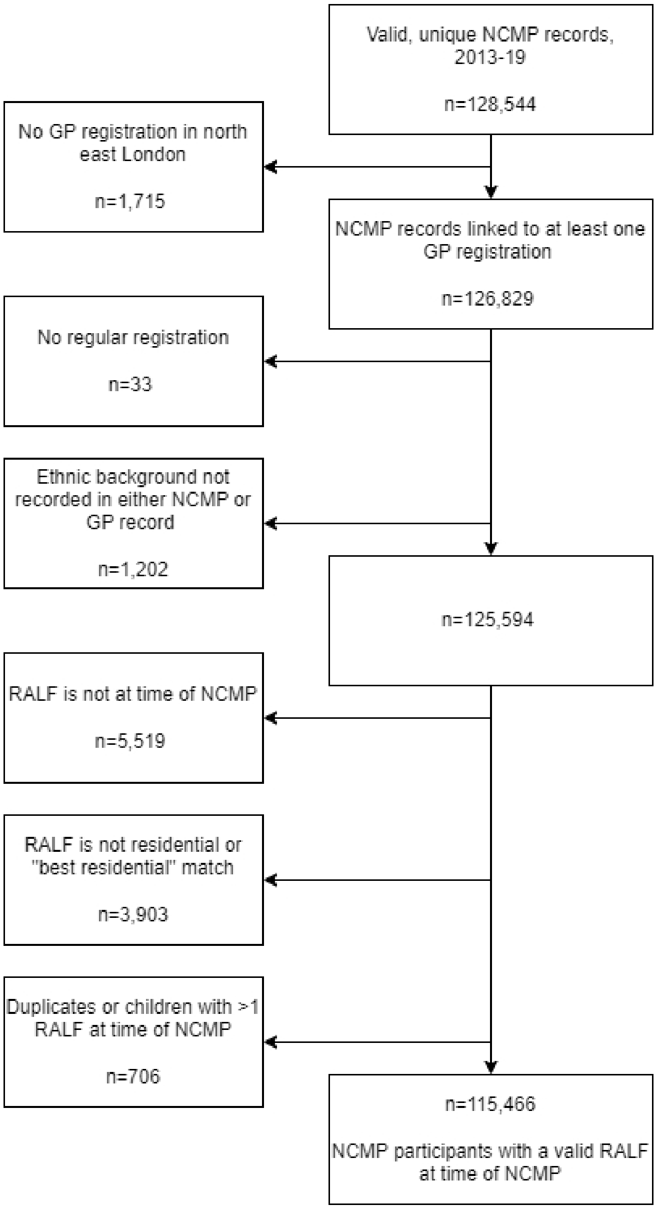

Identifying a valid Residential Anonymised Linkage Field (RALF) at the time of National Child Measurement Programme (NCMP) measurement

Children living in non-residential dwellings or where the UPRN match qualifier was not a "best" residential match were excluded (n=3,903). The match qualifier indicates how close to the place of residence the assigned UPRN is.

141x261mm (72 x 72 DPI)

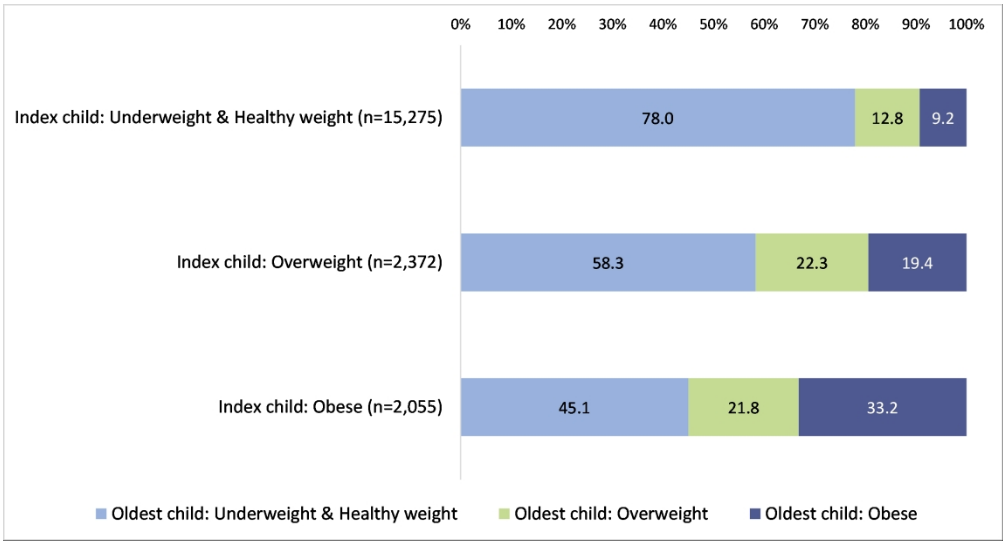

Oldest child's weight status stratified by index child's weight status[1]

[1] National Child Measurement Programme (NCMP)-recorded body mass index (BMI) after application of ethnic-specific BMI adjustments, categorised according to UK1990 clinical reference standard: "underweight or healthy weight" (<91st centile), "overweight" (≥91st to <98th centile), or "obese" (≥98th centile).

554x298mm (118 x 118 DPI)

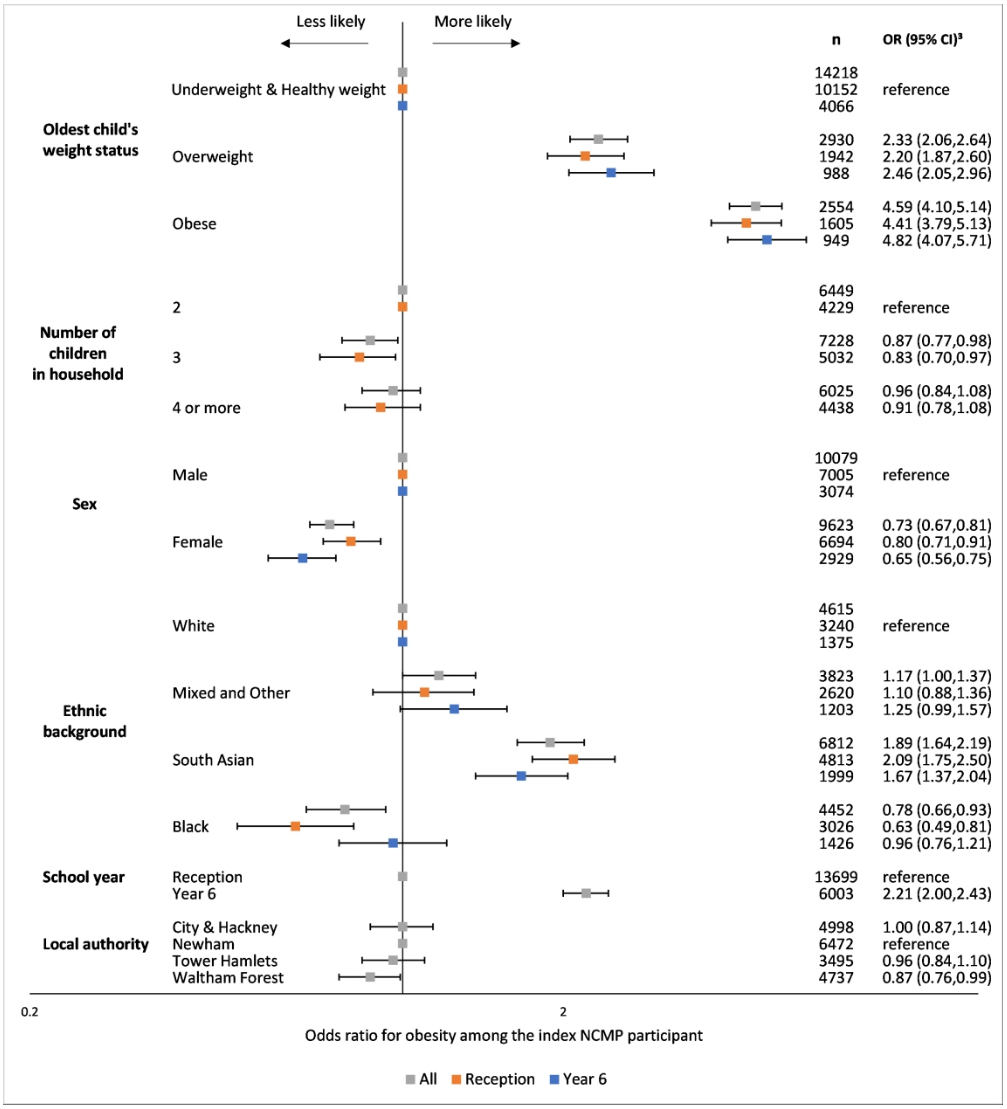

Multivariable[1] odds ratio estimating the likelihood of obesity[2] among the youngest children living in households with two National Child Measurement Programme participants

[1] The model including all households with two National Child Measurement Programme (NCMP) participants mutually adjusted for oldest child's weight status, number of children in the household, sex, ethnic background as recorded in the NCMP and, where missing, supplemented with ethnic background as recorded in the child's primary care electronic health record, school year of participation in the NCMP (Reception participants are aged 4-5 years and Year 6 participants are aged 10-11 years) and local authority of school where child participated in the NCMP. The model which only included households where the youngest child participated in the NCMP in Reception year mutually adjusted for the oldest child's weight status, number of children in the household, sex and ethnic background. The model which only included households where the youngest child participated in the NCMP in Year 6 mutually adjusted for the oldest child's weight status, sex and ethnic background. [2] NCMP-recorded body mass index (BMI) after application of ethnic-specific BMI adjustments, categorised according to UK1990 clinical reference standard: "obese" (≥98th centile). [3] Odds ratio and confidence interval. Odds ratios are plotted on a logarithmic scale.

577x635mm (118 x 118 DPI)

Is obesity more likely among children sharing a household with an older child with obesity? Cross-sectional study of linked National Child Measurement Programme data and electronic health records

Nicola Firman,[1] Marta Wilk,[1] Milena Marszalek,[1] Lucy Griffiths,[2] Gill Harper,[1] Carol Dezateux[1]

[1] Centre for Primary Care, Wolfson Institute of Population Health, Faculty of Medicine and Dentistry, Queen Mary University of London, Yvonne Carter Building, 58 Turner Street, London, E1 2AB

[2] Swansea University Medical School, Faculty of Medicine, Health & Life Sciences

Confidential: For Review Only

Supplementary material

Figure S1 – Relationship between address and General Practice (GP) registration dates and Residential Anonymised Linkage Fields (RALFs) at the time of National Child Measurement Programme (NCMP)

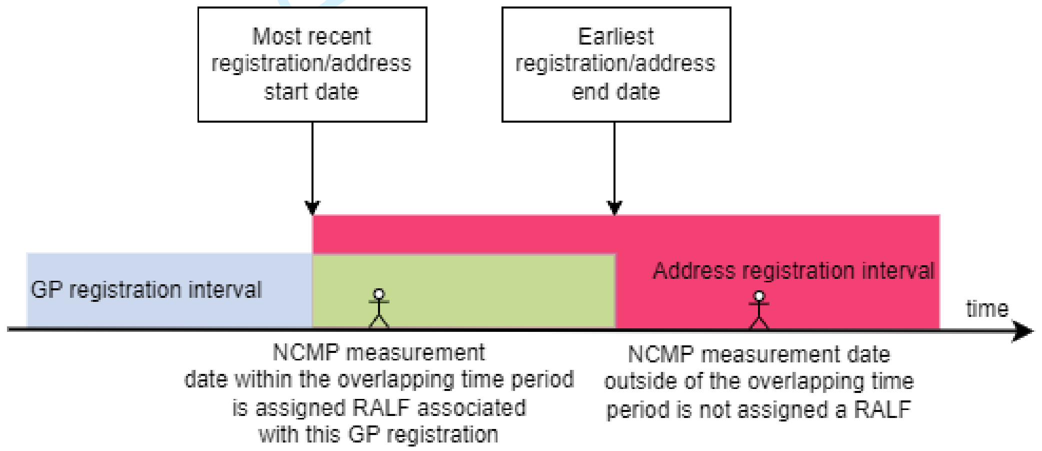

Figure S2 – Identifying National Child Measurement Programme (NCMP) participants' valid General Practice (GP)-recorded Residential Anonymised Linkage Fields (RALFs)

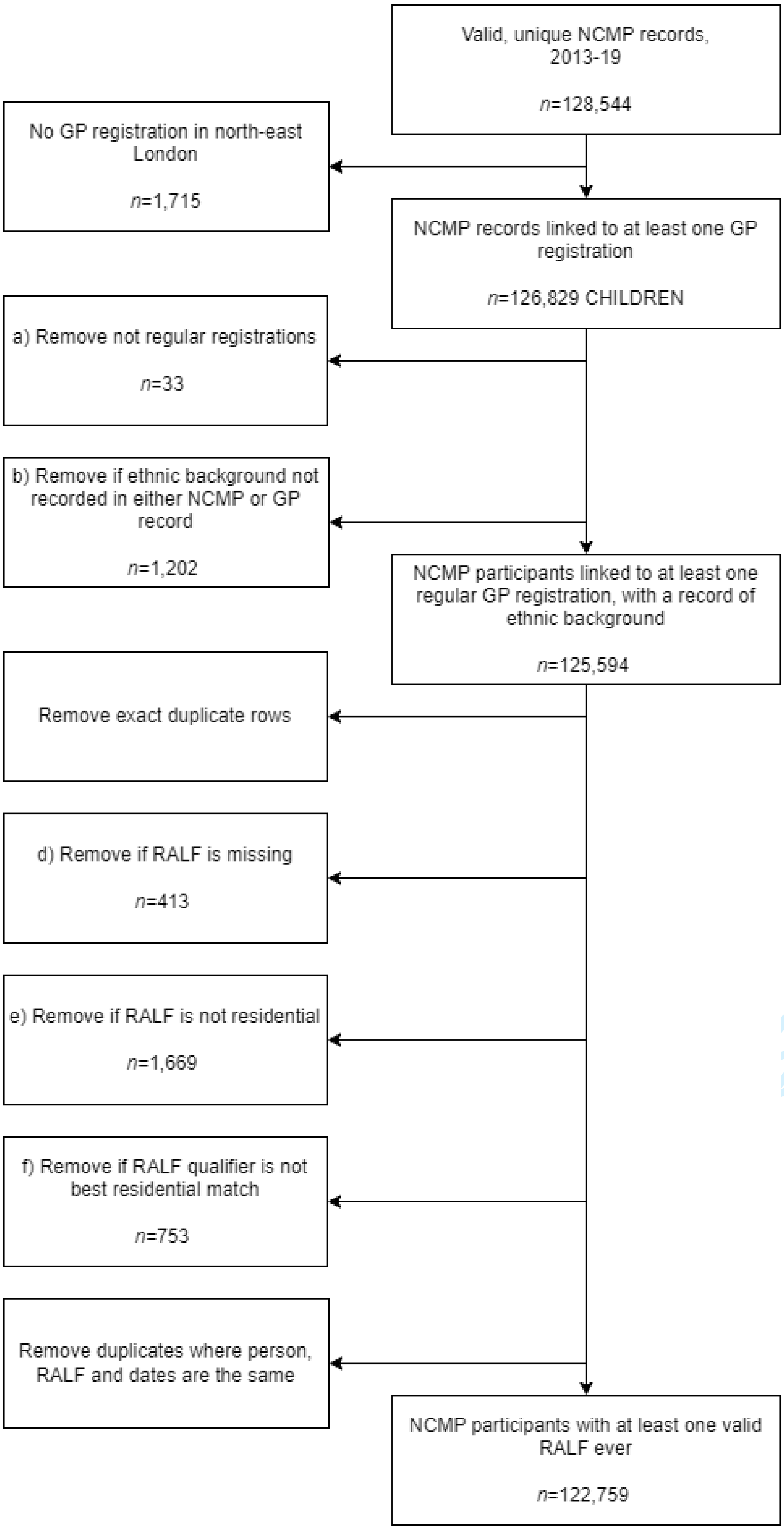

Figure S3 – Identifying National Child Measurement Programme (NCMP) participants sharing a household using Residential Anonymised Linkage Fields (RALFs)

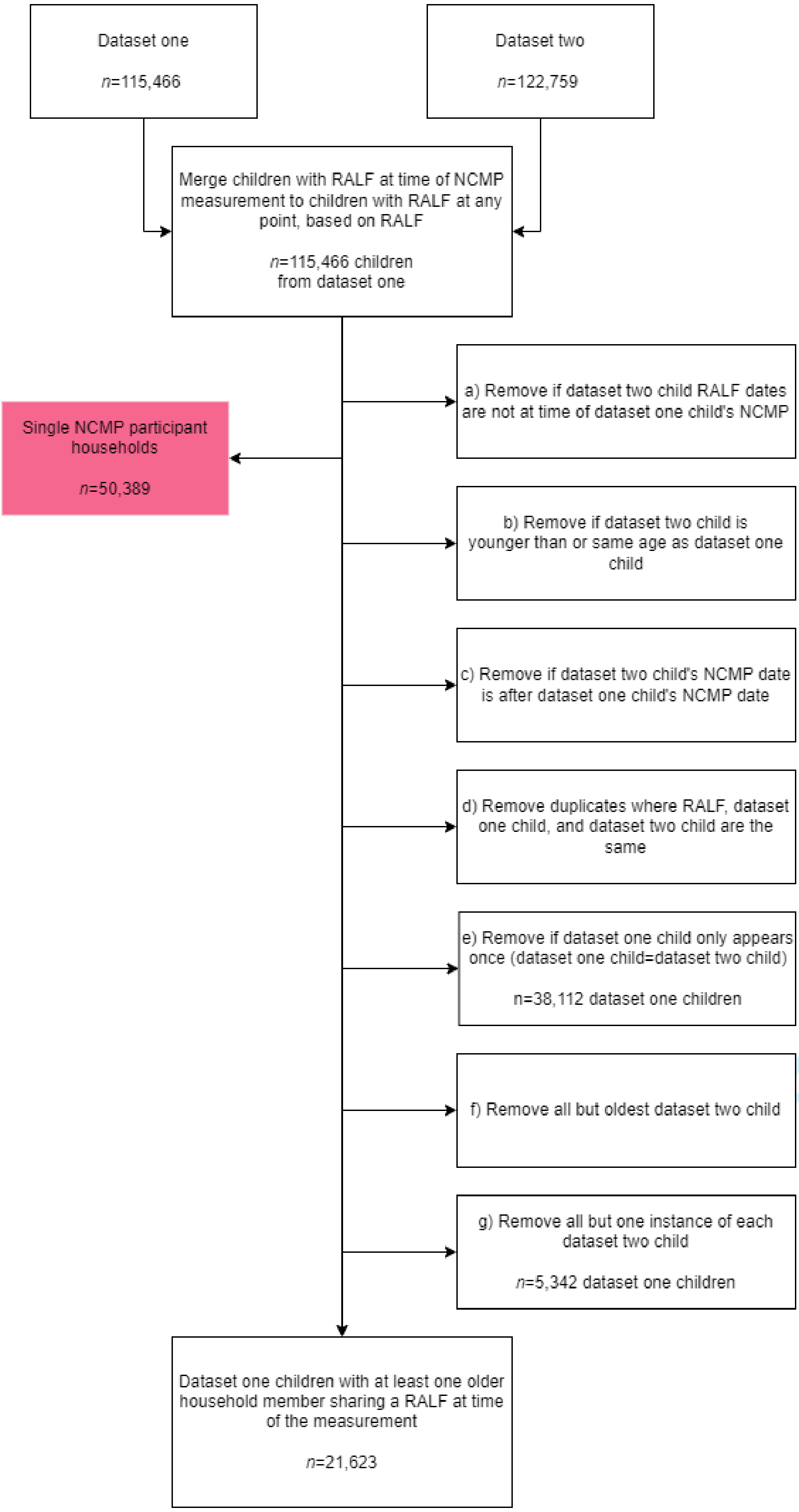

Index children were joined with children in dataset two using Stata's many to many `joinby` *function, combining all pairwise combinations on common RALFs. Where an older child matched to multiple index children, only one random pair was retained (n=5,342).*

Figure S4 – Identifying all other household members at the time of the index child's NCMP measurement

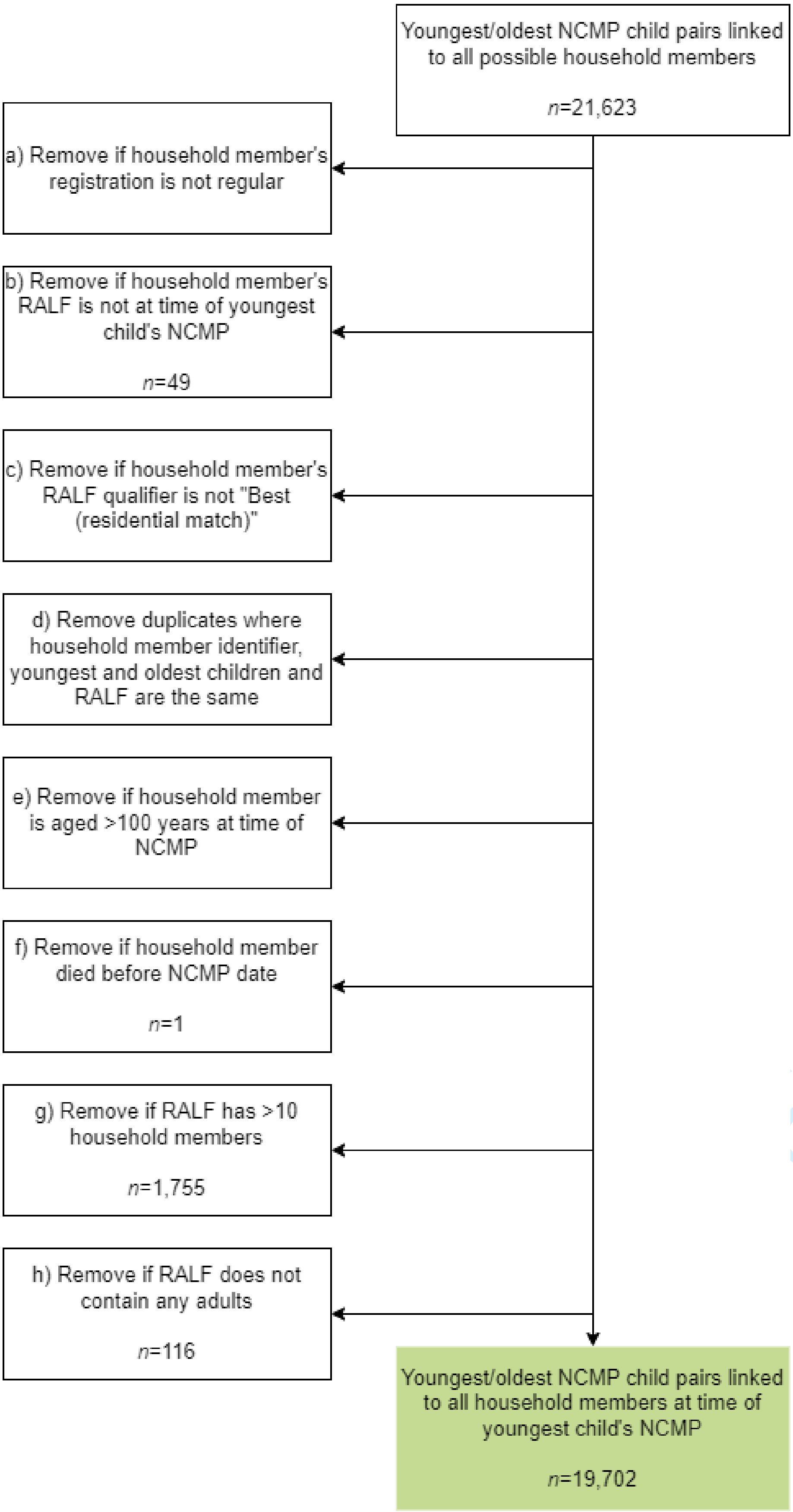

Only

For each household member, age at the time of the youngest child's NCMP measurement was calculated.

Table S1 – Sample characteristics of oldest National Child Measurement Programme participants in the household

The distribution by sex, ethnic background and weight status was broadly representative of NCMP participants in the four local authorities as a whole.

	**All (*n*=19,702)**			**Reception (*n*=6,506)**			**Year 6 (*n*=13,196)**		
	**n**	**%**	**95% CI[1]**	**n**	**%**	**95% CI[1]**	**n**	**%**	**95% CI[1]**
**Sex**									
Male	9975	50.6	49.9,51.3	3268	50.1	48.9,51.3	6707	50.9	50.0,51.7
Female	9727	49.4	48.7,50.1	3238	49.9	48.7,51.1	6489	49.1	48.3,50.0
**School year[2]**									
Reception	6506	33.0	32.3,33.6	6506	100.0				
Year 6	13196	67.0	66.4,67.7				13196	100.0	
**Academic year[3]**									
2013/14	3387	17.2	16.7,17.7	1418	21.8	20.8,22.9	1969	14.9	14.3,15.5
2014/15	3820	19.4	18.9,20.0	1412	21.7	20.7,22.7	2408	18.3	17.6,19.0
2015/16	5888	29.9	29.3,30.5	2075	31.9	30.8,33.0	3813	28.9	28.1,29.7
2016/17	1435	21.0	20.4,21.6	1251	19.2	18.3,20.2	2884	21.9	21.2,22.6
2017/18 & 2018/19	2472	12.5	12.1,13.0	350	5.3	4.9,6.0	2122	16.0	15.5,16.7
**Local authority[4]**									
City & Hackney	5041	25.6	25.0,26.2	1848	28.4	27.4,29.5	3193	24.2	23.5,24.9
Newham	3460	17.5	17.0,18.1	864	13.3	12.5,14.1	2596	19.6	19.0,20.3
Tower Hamlets	6458	32.8	32.1,33.5	2006	30.8	29.6,31.9	4452	33.8	33.0,34.6
Waltham Forest	4743	24.1	23.5,24.7	1788	27.5	26.5,28.6	2955	22.4	21.7,23.1
**Ethnic background[5]**									
White	4503	22.9	22.3,23.4	1873	28.8	27.7,29.9	2630	19.9	19.3,20.6
Mixed and Other	4118	20.9	20.3,21.5	1361	20.9	20.0,21.9	2757	20.9	20.2,21.6
South Asian	6857	34.8	34.1,35.5	1917	29.5	28.4,30.6	4940	37.4	36.6,38.3
Black	4224	21.4	20.9,22.0	1355	20.8	19.8,21.8	2869	21.7	21.1,22.5
**Weight status[6]**									
Underweight	280	1.4	1.3,1.6	97	1.5	1.2,1.8	183	1.4	1.2,1.6
Healthy weight	13938	70.7	70.1,71.4	5299	81.4	80.5,82.4	8639	65.5	64.7,66.3
Overweight	2930	14.9	14.4,15.4	679	10.4	9.7,11.2	2251	17.0	16.4,17.7
Obese	2554	13.0	12.5,13.4	431	6.7	6.1,7.3	2123	16.1	15.5,16.7

[1] Confidence interval. [2] School year of participation in the National Child Measurement Programme (NCMP), Reception participants are aged 4-5 years and Year 6 participants are aged 10-11 years. [3] Academic year of participation in the NCMP. Academic years run from September to July. The 2017/18 and 2018/19 academic years were combined to minimise risk of disclosure of individuals. [4] Local authority of school where child participated in the NCMP. [5] As recorded in the NCMP and, where missing, supplemented with ethnic background as recorded in the child's primary care electronic health record. [6] NCMP-recorded body mass index (BMI) after application of ethnic-specific BMI adjustments, categorised according to UK1990 clinical reference standard: "underweight" (BMI<2nd centile), "healthy weight" (≥2nd to <91st centile), "overweight" (≥91st to <98th centile), or "obese" (≥98th centile).

Confidential: For Review Only

Table S2 – Household characteristics of children living in households with two National Child Measurement Programme participants, by index child's weight status[1]

	Youngest child (*n*=19 702)								
	Underweight & Healthy weight (*n*=15 275)			Overweight (*n*=2 372)			Obese (*n*=2 055)		
**Number of children in the household**									
2	4 992	32.7	31.9,33.4	789	33.3	31.6,35.4	668	32.5	30.5,34.5
3	5 596	36.6	35.8,37.4	899	37.9	35.9,39.8	733	35.7	33.7,37.8
4 or more	4 687	30.7	30.0,31.5	684	28.8	26.9,30.6	654	31.8	29.8,33.8
**Household composition**									
Adults with children	11 593	75.9	75.2,76.6	1 797	75.8	74.0,77.5	1 586	77.2	75.3,78.9
Single adult with children	2 282	14.9	14.4,15.5	333	14.0	12.7,15.5	258	12.5	11.2,14.0
Three generation and skipped generation	1 400	9.2	8.7,9.6	242	10.2	9.0,11.5	211	10.3	9.1,11.7
**Property classification**									
Flat	7 887	51.6	50.8,52.4	1 253	52.8	50.8,54.8	1 120	54.5	52.3,56.6
Terraced house	6 375	41.7	41.0,42.5	970	40.9	38.9,42.9	809	39.4	37.3,41.5
Other	1 013	6.6	6.2,7.0	149	6.3	5.4,7.3	126	6.1	5.2,7.3

[1] National Child Measurement Programme (NCMP)-recorded body mass index (BMI) after application of ethnic-specific BMI adjustments, categorised according to UK1990 clinical reference standard: "underweight or healthy weight" (<91st centile), "overweight" (≥91st to <98th centile), or "obese" (≥98th centile). [2] Confidence interval.

Table S3 – Characteristics of index and oldest child pairs, by index child's weight status[1]

	Underweight & Healthy weight (*n*=15 275)			Overweight (*n*=2 372)			Obese (*n*=2 055)		
	n	%	95% CI[2]	n	%	95% CI[2]	n	%	95% CI[2]
**Oldest child's weight status[1]**									
Underweight	256	1.7	1.5,1.9	13	0.5	0.3,0.9	11	0.5	0.3,1.0
Healthy weight	11 652	76.3	75.6,76.9	1 371	57.8	55.7,59.7	915	44.5	42.5,46.8
Overweight	1 955	12.8	12.3,13.3	528	22.3	20.8,24.1	447	21.8	19.9,23.5
Obese	1 412	9.2	8.8,9.7	460	19.4	17.8,21.0	682	33.2	31.2,35.2
**Sex concordance[3]**									
Different	7 681	50.0	49.4,51.0	1 174	49.5	47.5,51.5	1 049	51.0	48.8,53.1
Same	7 594	49.7	49.0,50.6	1 198	50.5	48.5,52.5	1 006	49.0	46.9,51.2
**Time difference (months)[4]**									
Median (IQR[5])	23.3	12.6,33.1		23.4	12.6,33.0		22.9	12.5,31.9	

[1] National Child Measurement Programme (NCMP)-recorded body mass index (BMI) after application of ethnic-specific BMI adjustments, categorised according to UK1990 clinical reference standard: "underweight" (BMI<2nd centile), "healthy weight" (≥2nd to <91st centile), "overweight" (≥91st to <98th centile), or "obese" (≥98th centile). [2] Confidence interval. [3] Whether the youngest and oldest child were of the same or different sex. [4] Time difference, calculated in months, between the oldest and youngest child's NCMP measurements. [5] Interquartile range.

Confidential: For Review Only

Table S4 – Univariable and multivariable odds ratios estimating the likelihood of obesity[1] among index children living in households with two National Child Measurement Programme participants

	**All (*n*=19 702)**				**Reception (*n*=13 699)**				**Year 6 (*n*=6 003)**			
	**Univariable**		**Multivariable[2]**		**Univariable**		**Multivariable[3]**		**Univariable**		**Multivariable**	
	**OR[5]**	**95% CI[6]**	**OR[5]**	**95% CI[6]**	**OR[5]**	**95% CI[6]**	**OR[5]**	**95% CI[6]**	**OR[5]**	**95% CI[6]**	**OR[5]**	**95% CI[6]**
**Oldest child's weight status[7]**												
Underweight & Healthy weight (reference)	1		1		1		1		1		1	
Overweight	2.58	2.29,2.92	2.33	2.06,2.64	2.46	2.09,2.90	2.20	1.87,2.60	2.57	2.14,3.08	2.46	2.05,2.96
Obese	5.23	4.68,5.84	4.59	4.10,5.14	5.00	4.31,5.80	4.41	3.79,5.13	5.05	4.27,5.97	4.82	4.07,5.71
**Number of children in the household**												
2 (reference)	1		1		1		1		1			
3	0.98	0.87,1.09	0.87	0.77,0.98	1.00	0.86,1.17	0.83	0.70,0.97	1.03	0.87,1.21		
4 or more	1.05	0.94,1.18	0.96	0.84,1.08	1.13	0.97,1.32	0.91	0.78,1.08	1.14	0.96,1.35		
**Household composition**												
Adults with children (reference)	1				1				1			
Single adult with children	0.83	0.73,0.96			0.75	0.61,0.91			0.91	0.74,1.40		
Three and skipped generation	1.08	0.93,1.26			1.04	0.85,1.29			1.12	0.89,1.40		
**Sex**												
Male (reference)	1		1		1		1		1		1	
Female	0.74	0.68,0.81	0.73	0.67,0.81	0.81	0.71,0.92	0.80	0.71,0.91	0.66	0.58,0.76	0.65	0.56,0.75
**Ethnic background[8]**												
White (reference)	1		1		1		1		1		1	
Mixed and Other	1.25	1.07,1.45	1.17	1.00,1.37	1.13	0.91,1.40	1.10	0.88,1.36	1.36	1.08,1.70	1.25	0.99,1.57
South Asian	2.20	1.94,2.49	1.89	1.64,2.19	2.40	2.02,2.84	2.09	1.75,2.50	2.02	1.67,2.46	1.67	1.37,2.04
Black	0.84	0.71,0.98	0.78	0.66,0.93	0.61	0.48,0.78	0.63	0.49,0.81	1.04	0.83,1.31	0.96	0.76,1.21
**School year[9]**												
Reception (reference)	1		1									
Year 6	2.26	2.06,2.48	2.21	2.00,2.43								

Confidential: For Review Only

	All (*n*=19 702)				Reception (*n*=13 699)				Year 6 (*n*=6 003)			
	Univariable		Multivariable[2]		Univariable		Multivariable[3]		Univariable		Multivariable	
	OR[5]	95% CI[6]	OR[5]	95% CI[6]	OR[5]	95% CI[6]	OR[5]	95% CI[6]	OR[5]	95% CI[6]	OR[5]	95% CI[6]
**Academic year[10]**												
2013/14	0.70	0.32,1.52			0.86	0.37,1.99						
2014/15	0.83	0.60,1.16			1.01	0.69,1.49			1.17	0.58,2.34		
2015/16	0.94	0.80,1.11			1.16	0.95,1.43			0.93	0.70,1.24		
2016/17	0.91	0.80,1.03			1.01	0.85,1.21			0.94	0.78,1.15		
2017/18	0.90	0.81,1.01			0.93	0.79,1.08			0.94	0.80,1.11		
2018/19 (reference)	1				1				1			
**Local authority[11]**												
City & Hackney	0.74	0.66,0.84	1.00	0.87,1.14	0.67	0.57,0.80			0.84	0.70,1.01		
Newham (reference.)	1		1		1				1			
Tower Hamlets	1.15	1.02,1.30	0.96	0.84,1.10	1.25	1.06,1.47			1.13	0.92,1.38		
Waltham Forest	0.74	0.65,0.84	0.87	0.76,0.99	0.69	0.58,0.83			0.76	0.63,0.92		
**Sex concordance[12]**												
Different (reference.)	1				1				1			
Same	0.97	0.88,1.06			0.96	0.85,1.08			1.00	0.87,1.14		
**Time difference (months)[13]**												
	1.00	0.99,1.00			0.99	0.99,1.00			1.00	0.99,1.00		

[1] National Child Measurement Programme (NCMP)-recorded body mass index (BMI) after application of ethnic-specific BMI adjustments, categorised according to UK1990 clinical reference standard: "obese" (≥98th centile). [2] Mutually adjusting for oldest child's weight status, number of children in the household, sex, ethnic background and school year. [3] Mutually adjusting for the oldest child's weight status, number of children in the household, sex and ethnic background. [4] Mutually adjusting for the oldest child's weight status, sex and ethnic background. [5] Odds ratio. [6] Confidence interval. [7] National Child Measurement Programme (NCMP)-recorded body mass index (BMI) after application of ethnic-specific BMI adjustments, categorised according to UK1990 clinical reference standard: "underweight or healthy weight" (<91st centile), "overweight" (≥91st to <98th centile), or "obese" (≥98th centile). [8] As recorded in the NCMP and, where missing, supplemented with ethnic background as recorded in the child's primary care electronic health record. [9] School year of participation in the National Child Measurement Programme (NCMP), Reception participants are aged 4-5 years and Year 6 participants are aged 10-11 years. [10] Academic year of participation in the National Child Measurement Programme (NCMP). Academic years run from September to July. [11] Local authority of school where child participated in the NCMP. [12] Whether the youngest and oldest child were of the same or different sex. [13] Time difference, calculated in months, between the oldest and youngest child's NCMP measurements. Cells highlighted in bold indicate occasions where the estimates are significantly difference to the reference group in the multivariable models.

Confidential: For Review Only

BMJ Paediatrics Open

Is obesity more likely among children sharing a household with an older child with obesity? Cross-sectional study of linked National Child Measurement Programme data and electronic health records

Journal:	*BMJ Paediatrics Open*
Manuscript ID	bmjpo-2024-002533.R1
Article Type:	Original research
Date Submitted by the Author:	25-Mar-2024

Complete List of Authors:	Firman, Nicola; Queen Mary University of London, Wolfson Institute of Population Health<br>Wilk, Marta; Queen Mary University of London, Wolfson Institute of Population Health<br>Marszalek, Milena; Queen Mary University of London, Wolfson Institute of Population Health<br>Griffiths, Lucy; Swansea University, Faculty of Medicine, Health & Life Sciences<br>Harper, Gill; Queen Mary University of London, Wolfson Institute of Population Health<br>Dezateux, Carol; Queen Mary University of London, Wolfson Institute of Population Health

Keywords:	Obesity, Epidemiology

SCHOLARONE™
Manuscripts

I, the Submitting Author has the right to grant and does grant on behalf of all authors of the Work (as defined in the below author licence), an exclusive licence and/or a non-exclusive licence for contributions from authors who are: i) UK Crown employees; ii) where BMJ has agreed a CC-BY licence shall apply, and/or iii) in accordance with the terms applicable for US Federal Government officers or employees acting as part of their official duties; on a worldwide, perpetual, irrevocable, royalty-free basis to BMJ Publishing Group Ltd ("BMJ") its licensees and where the relevant Journal is co-owned by BMJ to the co-owners of the Journal, to publish the Work in this journal and any other BMJ products and to exploit all rights, as set out in our licence.

The Submitting Author accepts and understands that any supply made under these terms is made by BMJ to the Submitting Author unless you are acting as an employee on behalf of your employer or a postgraduate student of an affiliated institution which is paying any applicable article publishing charge ("APC") for Open Access articles. Where the Submitting Author wishes to make the Work available on an Open Access basis (and intends to pay the relevant APC), the terms of reuse of such Open Access shall be governed by a Creative Commons licence – details of these licences and which Creative Commons licence will apply to this Work are set out in our licence referred to above.

Other than as permitted in any relevant BMJ Author's Self Archiving Policies, I confirm this Work has not been accepted for publication elsewhere, is not being considered for publication elsewhere and does not duplicate material already published. I confirm all authors consent to publication of this Work and authorise the granting of this licence.

Confidential: For Review Only

Is obesity more likely among children sharing a household with an older child with obesity? Cross-sectional study of linked National Child Measurement Programme data and electronic health records

Nicola Firman (ORCiD 0000-0001-5213-5044),[1] Marta Wilk,[1] Milena Marszalek,[1] Lucy Griffiths,[2] Gill Harper,[1] Carol Dezateux[1]

[1] Centre for Primary Care, Wolfson Institute of Population Health, Faculty of Medicine and Dentistry, Queen Mary University of London, Yvonne Carter Building, 58 Turner Street, London, E1 2AB

[2] Swansea University Medical School, Faculty of Medicine, Health & Life Sciences

Corresponding author: Nicola Firman; Centre for Primary Care, Wolfson Institute of Population Health, Faculty of Medicine and Dentistry, Queen Mary University of London, Yvonne Carter Building, 58 Turner Street, London, E1 2AB; Nicola.firman@qmul.ac.uk; 0207 882 6806

The authors declare no competing financial interests.

Abstract

Background/objectives

We identified household members from electronic health records linked to National Child Measurement Programme (NCMP) data to estimate the likelihood of obesity among children living with an older child with obesity.

Methods

We included 126,829 NCMP participants in four London boroughs and assigned households from encrypted Unique Property Reference Numbers for 115,466 (91.0%). We categorised the ethnic-adjusted body mass index of the youngest and oldest household children (underweight/healthy weight<91st, ≥91st overweight<98th, obesity≥98th centile) and estimated adjusted odds ratios (OR) and 95% confidence intervals (CI) of obesity in the youngest child by the oldest child's weight status, adjusting for number of household children (two, three or ≥4), youngest child's sex, ethnicity, and school year of NCMP participation.

Results

We identified 19,702 households shared by two or more NCMP participants (% male; median age, range (years), youngest children: 51.2%; 5.2, 4.1-11.8; oldest children: 50.6%; 10.6, 4.1-11.8). One third of youngest children with obesity shared a household with another child with obesity (33.2%; 95% CI: 31.2,35.2), compared with 9.2% (8.8,9.7) of youngest children with a healthy weight. Youngest children living with an older child considered overweight (OR: 2.33; 95% CI: 2.06,2.64) or obese (4.59, 4.10,5.14) were more likely to be living with obesity.

Conclusions

Identifying children sharing households by linking primary care and school records provides novel insights into the shared weight status of children sharing a household. Qualitative research is needed to understand how food practices vary by household characteristics to increase understanding of how the home environment influences childhood obesity.

Confidential: For Review Only

Key words

Childhood, obesity, households, BMI, electronic health records, record linkage

Key messages

There is evidence to suggest that children living with older siblings with obesity are more likely to be living with obesity themselves. Research to date has largely focused on the weight status of biological siblings .

What this study adds

We examined associations between child household weight status using novel linkages between school measurement and electronic health records. We showed that younger children living with an older child with obesity were more than four times more likely to live with obesity than those living with an older child with a healthy weight.

How this study might affect research, practice or policy

Household factors are potentially more modifiable than genetic or prenatal influences. Taking a household-level approach could potentially reach more children living with, and at risk of, obesity.

Confidential: For Review Only

Introduction

Childhood obesity is a major public health concern globally and reflects a complex number of factors, in particular socio-economic inequalities.(1) In England, more than one quarter of children leave primary school with overweight or obesity at a level of severity defined as in need of clinical intervention.(2)

A child's health, including their weight status, is significantly affected by the environment in which they live. Better understanding of households, their composition, and the health of children and adults who share households may provide novel actionable insights to address unhealthy weight in childhood.

Research has shown that child obesity is associated with parental obesity, where parental overweight or obesity is associated with an increased risk of obesity in their child.(3) This relationship is stronger for mothers than fathers.(4, 5) Less is known about the associations between the obesity status of child household members. Research investigating associations between siblings' weight status has reported inconsistent results.(6-13) Children living together may experience similar genetic, environmental, and socioeconomic circumstances, which may in turn contribute to a shared risk for obesity. The shared household environment is potentially more modifiable than genetic or prenatal influences.

A 2023 systematic review identified that siblings' health-related behaviours and weight-related outcomes varied according to sibling sex and birth order.(14) Our understanding of how household composition, including presence of a sibling or other household children, as well as their weight status, influences childhood obesity could be improved with further research which includes all child household members and not just those who are biologically-related.

We identified individuals sharing a household using electronic health records and linked this to school measurement programme data to estimate the likelihood of obesity among children living with an older child with obesity. We hypothesised that younger children will be more likely to be living with obesity if they share a household with an older child living with obesity. We also investigated whether household composition and size, and dwelling type, influenced the likelihood of childhood obesity.

Confidential: For Review Only

Methods

Study population

Children in the first (Reception year) and last (Year 6) years of primary school are invited to participate in the National Child Measurement Programme (henceforth known as the school measurement programme), which measures the height and weight of 4-5- and 10-11-year-olds attending state-maintained schools in England. More than one million children take part annually, with participation rates remaining higher than 90% since 2010/11.(15) School participation is voluntary, although over 99% participate.(16) In City & Hackney, approximately one quarter of school-aged children attend private or faith schools, compared with equivalent figures of 1.4%, 5.0% and 5.0% for Newham, Tower Hamlets, and Waltham Forest, respectively.(17) We do not have information about the small proportion of children who opt out of the school measurement programme.

We linked 126,829 of 128,544 (98.7%) school measurement programme records from four north-east London local authorities (City & Hackney, Newham, Tower Hamlets, and Waltham Forest) to general practice (GP) electronic health records via the Discovery Data Service.(18)

Data sources

We obtained pseudonymised school measurement programme data for the 2013/14-2018/19 academic years under data processing agreements with each local authority public health department. We only received school measurement programme records that had been returned to each local authority after quality assurance checks.(19) As the available date of school measurement programme measurement was restricted to month and year, we randomly assigned a day of measurement within term-time, excluding weekends and bank holidays to avoid a spurious reduction in variance in age at measurement occasioned by using the same fixed date of measurement for every child (R Studio; version 1.0.153; code available here: bit.ly/random_day).

Pseudonymised data were provided from the Discovery Data Service which receives primary care electronic health records on a daily basis from all GPs in north-east London. Demographic and clinical data recorded up to 1st November 2021 were extracted for school measurement programme participants successfully linked to the Discovery Data Service via pseudonymised NHS numbers created using OpenPseudonymiser software.(20)

Confidential: For Review Only

All data were extracted and managed according to UK National Health Service (NHS) information governance requirements.(21)

Data processing

Residential Anonymised Linkage Fields

Every addressable location in Great Britain is assigned a Unique Property Reference Number (UPRN). UPRNs identify a place of residence at a granular level, identifying individual properties, for example houses or flats within a block or building shell. UPRNs are allocated to GP-recorded addresses using the validated ASSIGN algorithm,(22) and pseudonymised into Residential Anonymised Linkage Fields (RALFs) within the Discovery Data Service, using a study-specific encryption key.

Identifying household members at the child's school measurement date

A household can only be defined at a single point in time because people living at an address may change over time whilst the UPRN assigned to the residential dwelling stays the same.

A data extract containing all RALFs associated with any address(es) recorded in a child's electronic health record was extracted. The file contained start and end dates of patient registration (enrolment) with the GP as well as address start and end dates. Address start and end dates refer to the dates at which a patient lived at a particular address. In most cases, these align with GP registration dates, but could differ, if for example, a patient moved house but remained registered with the same GP.

Figure 1 describes the process for deciding which, if any, of the child's RALFs was the place of residence at the time of their school measurement programme measurement. If the school measurement programme date of measurement took place between the most recent of the registration and address start dates and the earliest of the registration and address end dates, the RALF was considered to be the place of residence at the time of school measurement programme (supplementary Figure S1). Children without a RALF at the time of their school measurement programme were excluded (*n*=5,519). We retained 115,466 children with a RALF at the time of their school measurement programme measurement (referred to hereafter as index children).

Confidential: For Review Only

Identifying other National Child Measurement Programme participants in the household

Similar steps were taken to identify other school measurement programme participants sharing a household with index children. We started with 122,759 school measurement programme participants with at least one RALF (supplementary Figure S2) and included in "dataset two".

Child pairs were excluded if the index child's school measurement programme measurement date did not fall within dataset two child's RALF dates (supplementary Figure S3). Pairs were also excluded if the dataset two child was younger than the index child or if the dataset two child's school measurement programme measurement was after the index child's. This process found the youngest index child and identified the oldest school measurement programme participant living in the same household at the time of the index child's school measurement programme measurement. Of 128,554 school measurement programme participants, 21,623 youngest/oldest child pairs were identified.

Identifying other household members and deriving household characteristics

We identified all people who had ever lived at any of the RALFs identified in the dataset of youngest/oldest child pairs. Steps were taken to determine household members at the time of the index child's school measurement programme measurement (supplementary Figure S4). Full household information was identified for 19,702 youngest/oldest child pairs.

Outcome of interest

Obesity status of the index child was defined by the UK1990 clinical reference standard.(23) After application of ethnic-specific BMI adjustments,(24) a binary variable indicating obesity was defined as a BMI greater than or equal to the 98th age- and sex-specific centile. The index child's ethnic-adjusted BMI *z*-score was a secondary outcome.

Explanatory variables

Ethnic-specific BMI adjustments(24) were applied to the older child's BMI, and weight status determined according to the UK1990 clinical reference standard(23) categorising BMI into one of four mutually exclusive groups: "underweight" (<2nd centile), "healthy weight" (≥2nd to <91st centile), "overweight" (≥91st to <98th

https://mc.manuscriptcentral.com/bmjpo

centile) or “obese” (≥98th centile). The older child’s BMI *z*-score was also considered as an explanatory variable.

School measurement programme -recorded sex, local authority of the school where the child participated in the school measurement programme, academic year (September to July) and school year (Reception/Year 6) of participation in the school measurement programme were explanatory variables.

School measurement programme -recorded ethnic background was grouped into four mutually exclusive groups(25): White (‘White British’, ‘White Irish’, or ‘any other White background’); Black (‘Black African’, ‘Black Caribbean’, or ‘any other Black background’); South Asian (‘Indian’, ‘Pakistani’, ‘Bangladeshi’ or ‘Sri Lankan’); and a combination of Mixed and Other (‘any other ethnic background’, ‘mixed ethnicity’, ‘Chinese’ or ‘Asian other’). Where ethnic background was missing or not stated in the school measurement programme, ethnic background as recorded in the electronic health record (n=11,077) was used.

An area-level measure of relative deprivation - Index of Multiple Deprivation (IMD) decile(26) - was assigned to each school measurement programme record based on the postcode of the child’s home address. IMD decile was concatenated into five quintiles ranging from most to least deprived.

A categorical variable was derived from a count of children (aged <18.0 years) assigned the same RALF as the school measurement programme participant, grouped as follows: 2; 3-4; 5-6; 7-10.

We categorised household composition using a modified Harper and Mayhew method(27) by counting the number of household members in three age brackets: 0-17 years (children), 18-64 years (working age adults) and 65 or older (older adults) and grouping into: working-aged adults with children; a single working-age adult with children; at least one working-age and one older adult with children (three generation household), or at least one older adult with children (skipped generation household).

The property classification, as given by the ASSIGN algorithm, categorised properties into three groups: flats, terraced houses, and other.

Sex concordance was coded either the same (when both children shared the same sex) or different (when the two children had differing sexes). The time difference between the youngest and oldest children’s school

Confidential: For Review Only

measurement programme measurements was calculated as the time in months between the two measurements.

Statistical analyses

We estimated the prevalence of obesity among children living with an older school measurement programme participant and explored variation by the weight status of the older child. We used binary logistic regression to estimate the likelihood of obesity in the index child (odds ratio [OR] and 95% confidence interval [CI]) by the older child's weight status, after accounting for individual and household characteristics. We conducted linear regression to estimate the effect of a one unit increase in the oldest child's BMI *z*-score on the index child's BMI *z*-score, after checking residuals were normally distributed. All analyses, conducted using Stata (MP/15.0), were stratified by school year.

Patient and public involvement

This research was done without patient or public involvement. Neither were invited to comment on the study design and were not consulted to develop relevant outcomes or interpret results.

Ethics approval

The analyses of linked pseudonymised school measurement programme and GP data were approved by the respective data controllers under data processing agreements which allow linkage of pseudonymised school measurement programme data between the research organisation and each local authority public health team. This study is a secondary analysis of de-identifiable data and no further ethics approval was required.

Results

Index children were, by study design, more likely to take part in the school measurement programme in the Reception school year and in the more recent academic years (Table 1). Similarly, the oldest children were more likely to take part in the school measurement programme in Year 6 and in the earlier academic years (supplementary Table S1). 7.9% of Reception year youngest children and 16.3% of Year 6 youngest children were living with obesity (Table 1). Equivalent estimates using International Obesity Task Force cut-offs are

Confidential: For Review Only

reported in supplementary Table S2. Three quarters lived in households with adults of working age only, and more than half lived in flats (Table 2).

A greater proportion of index children with obesity were male, participating in the school measurement programme in Year 6 and in Tower Hamlets and Newham, and from South Asian ethnic backgrounds, compared with index children with underweight or a healthy weight (Table 3).

There was no variation in the number of children sharing a household or property classification by weight status (Table S3). A lower proportion of index children living with obesity lived in households with a single adult (12.5%; 95% CI: 11.2,14.0) compared with the proportion among children with an underweight/healthy weight status (14.9%; 14.4,15.5).

One fifth and one third of index children living with obesity shared a household with another child with overweight or obesity, respectively, higher than those with underweight or of a healthy weight (Table S4; Figure 2). Sex concordance, nor time difference between the index and older children's school measurement programme measurement dates, did not vary by weight status of the index child.

In adjusted analyses, index children living with an older child with overweight or obesity were more likely to be living with obesity. Conversely, those sharing a household with two other children were less likely to be living with obesity (Figure 3; uni- and multi-variable results are presented in Table S5).

In multivariable linear regression, a one unit increase in the oldest child's BMI *z*-score was associated with a 0.32 (95% CI: 0.30,0.33) increase in the index child's BMI *z*-score. Similarly, in linear regression models stratified by the school year of participation in the school measurement programme, a one unit increase in the oldest child's BMI *z*-score predicted a 0.28 (0.27,0.29) and 0.38 (0.35,0.40) increase in Reception and Year 6 index child's BMI *z*-scores, respectively.

Discussion

Summary of key findings

We examined associations between child household weight status using novel linkages between school measurement and electronic health records. We showed that younger children living with an older child with

Confidential: For Review Only

obesity were more than four times more likely to live with obesity than those living with an older child with a healthy weight.

Strengths and limitations

We used UK1990 clinical thresholds to identify children with obesity considered in need of clinical intervention, as advised by the Scientific Advisory Committee on Nutrition,(23) in an ethnically-diverse area of London with high levels of childhood obesity. We recognise these cut-offs do not allow for international comparisons. Our findings may not be generalisable to areas in the UK with lower levels of deprivation and ethnic diversity. The school measurement programme has high participation rates, but our study sample did not include children attending non-state maintained schools of which there is a higher proportion in City & Hackney.

We used linked school measurement programme records of weight status as we have previously shown that GP electronic health records do not contain accurate, up-to-date child measurement data and are biased to children at both extremes of the BMI distribution.(28) This resulted in exclusion of 30,552 school measurement programme participants who did not live with another school measurement programme participant in the 2013-2019 academic years.

We used a robust methodology to identify household members at the time of the school measurement programme measurement. The ASSIGN algorithm has been shown to match 98.6% of primary care patient addresses to UPRNs.(22) We adopted a conservative approach to identify "true" household members, by excluding school measurement programme participants living in large or non-residential households. It is possible that we included patients who no longer live at their registered address (so would not consult with their registered GP). There is also likely to be a time lag between a patients' GP registrations, and a period of time where a patient has moved on from an area but remains registered with a GP. Hence, we may have overestimated the true number of household members.

We were not able to determine whether child household members were biologically related. Similarly, we were not able to identify biological parents and account for parental BMI in our analyses.

Comparison with existing literature

https://mc.manuscriptcentral.com/bmjpo

Confidential: For Review Only

Our findings support those reporting an increased likelihood of obesity among children living with other children with obesity.(12, 14) There are likely to be several explanations for this. Firstly, children in the same household spend their time together and share the same resources, which supports the notion of the 'shared home environment'.(29) Siblings eat similar diets, and participate in similar levels of physical activity and sedentary behaviours.(14) Others note that older children are important influencers in children's health-related behaviours, particularly healthy eating. Younger children want to copy the behaviours of their older siblings, explaining the positive correlation between both children's healthy and unhealthy behaviours.(30, 31) Children living in the same household are likely to be exposed to the same level of family income, and potentially the same diet and physical activity.(32) Outside of the home, children will be exposed to the same built environment. Finally, biologically related children sharing the same household may share a common genetic predisposition to obesity.(33)

Implications for research, policy and practice

Our findings highlight the importance of understanding the household distribution of childhood obesity when designing services in populations with high prevalences of obesity and limited resources. A household-level approach may be a pragmatic response to identifying higher risk households by considering information about all resident children. The shared household environment is potentially more modifiable than genetic or prenatal influences, and analyses of the shared weight status of household members provides new insights about people sharing the same living space, regardless of their biological relationships. This insight is particularly pertinent now that children are increasingly living with household members with whom they may have no biological relationship.(34)

Routinely collected electronic health records provide a limited view of the home environment, and further qualitative research is necessary to fully understand who the decision-makers are, and how practices and attitudes relating to food purchasing and diet, as well as physical activity opportunities, are negotiated on a daily basis.

https://mc.manuscriptcentral.com/bmjpo

Confidential: For Review Only

Conclusion

Children living with an older child with obesity are more likely to be living with obesity. Early intervention should be approached from a household perspective which takes into account the roles of, and implications for, all household members.

Confidential: For Review Only

Funding

This work was supported by ADR UK (Administrative Data Research UK), an Economic and Social Research Council investment (part of UK Research and Innovation) [Grant number: ES/X00046X/1]. This research was also supported by grants from Barts Charity ref: MGU0419 and MGU0504. This work was supported by the UK Prevention Research Partnership (MR/S037527/1), which is funded by the British Heart Foundation, Cancer Research UK, Chief Scientist Office of the Scottish Government Health and Social Care Directorates, Engineering and Physical Sciences Research Council, Economic and Social Research Council, Health and Social Care Research and Development Division (Welsh Government), Medical Research Council, National Institute for Health Research, Natural Environment Research Council, Public Health Agency (Northern Ireland), The Health Foundation and Wellcome.

Competing interests

The authors declare no competing financial interests.

Author contributions

CD obtained funding for the study. NF, CD and GH conceptualised and designed the analyses. NF, MW, MM, GH and CD contributed to the development of the methodology. NF carried out the literature search, conducted the analyses, generated tables and figures and drafted the initial manuscript. NF, MW, MM, GH, LG, and CD contributed to the interpretation of analyses and reviewed and revised the manuscript. NF, MW, MM, GH, LG, and CD were involved in writing the paper and had final approval of the submitted and published manuscript. The corresponding author attests that all listed authors meet authorship criteria and that no others meeting the criteria have been omitted.

Data sharing

Access to primary care data is enabled by data sharing agreements between the Discovery Data Service and the data controllers. The Discovery Programme Board has approved data access by the REAL Child Health programme team for research on the condition that it is not onwardly shared. National Child Measurement Programme data were accessed under data processing agreements with each of the local authorities as data controllers in line with Public Health England guidance. These agreements preclude onward sharing of data.

Acknowledgements

The authors are grateful to the local authority public health teams for providing pseudonymised National Child Measurement Programme data, school nurses and National Child Measurement Programme data collection teams, and National Child Measurement Programme participants. We are grateful to Professor Mario Cortina-Borja (University College London Great Ormond Street Institute of Child Health) for supporting the development of an algorithm to randomly assign a day of measurement. We are grateful to the data controllers of the Discovery Programme and to the GPs and their practice teams for allowing the use of their patient records, to the Clinical Effectiveness Group for providing access to their curated high-quality dataset, and to the population in east London from whom the data are derived. This work uses data provided by patients and collected by the NHS as part of their care and support.

Confidential: For Review Only

Tables

Table 1 – Sample characteristics of index children participating in the National Child Measurement Programme

	All (*n*=19,702)			Reception (*n*=13,699)			Year 6 (*n*=6,003)		
	n	%	95% CI[1]	n	%	95% CI[1]	n	%	95% CI[1]
**Sex**									
Male	10079	51.2	50.5,51.9	7005	51.2	50.4,52.1	3074	51.1	49.9,52.4
Female	9623	48.8	48.1,49.5	6694	48.8	47.9,49.6	2929	48.9	47.6,50.1
**School year[2]**									
Reception	13699	69.5	68.9,70.2	13699	100.0				
Year 6	6003	30.5	29.8,31.1				6003	100.0	
**Academic year[3]**									
2031/14 & 2014/15	517	2.6	2.4,2.9	463	3.4	3.1,3.7	54	0.9	0.6,1.2
2015/16	1926	9.8	9.4,10.2	1530	11.2	10.7,11.7	396	6.6	6.0,7.3
2016/17	3751	19.0	18.5,19.6	2748	20.0	19.4,20.7	1003	16.7	15.8,17.7
2017/18	5980	30.4	29.7,31.0	4100	30.0	29.2,30.7	1880	31.3	30.1,32.5
2018/19	7528	38.2	37.5,38.9	4858	35.5	34.7,36.3	2670	44.5	43.2,45.7
**Local authority[4]**									
City & Hackney	4998	25.4	24.8,26.0	3489	25.5	24.7,26.2	1509	25.2	24.1,26.3
Newham	6472	32.9	32.2,33.5	4444	32.5	31.7,33.3	2028	33.8	32.6,35.0
Tower Hamlets	3495	17.7	17.2,18.3	2571	18.7	18.1,19.4	924	15.4	14.5,16.3
Waltham Forest	4737	24.0	23.4,24.6	3195	23.3	22.6,24.0	1542	25.6	24.6,26.8
**Ethnic background[5]**									
White	4615	23.4	22.9,24.0	3240	23.7	22.9,24.4	1375	22.9	21.9,24.0
Mixed and Other	3823	19.4	18.8,19.9	2620	19.1	18.4,19.8	1203	20.0	19.0,21.1
South Asian	6812	34.6	33.9,35.3	4813	35.1	34.3,35.9	1999	33.3	32.1,34.5
Black	4452	22.6	22.0,23.2	3026	22.1	21.4,22.8	1426	23.7	22.7,24.8
**Weight status[6]**									
Underweight	270	1.4	1.2,1.5	194	1.4	1.2,1.6	76	1.3	1.0,1.6
Healthy weight	15005	76.2	75.6,76.8	11025	80.5	79.9,81.2	3980	66.3	65.1,67.5
Overweight	2372	12.0	11.5,12.4	1399	10.2	9.7,10.7	973	16.1	15.2,17.0
Obese	2055	10.4	10.0,10.9	1081	7.9	7.4,8.3	974	16.3	15.4,17.3

[1] Confidence interval. [2] School year of participation in the National Child Measurement Programme (NCMP), Reception participants are aged 4-5 years and Year 6 participants are aged 10-11 years. [3] Academic year of participation in the NCMP. Academic years run from September to July. The 2013/14 and 2014/15 academic years were combined to minimise the risk of disclosing individuals. [4] Local authority of school where child participated in the NCMP. [5] As recorded in the NCMP and, where missing, supplemented with ethnic background as recorded in the child's primary care electronic health record. [6] NCMP-recorded body mass index (BMI) after application of ethnic-specific BMI adjustments, categorised according to UK1990 clinical reference standard: "underweight" (BMI<2nd centile), "healthy weight" (≥2nd to <91st centile), "overweight" (≥91st to <98th centile), or "obese" (≥98th centile).

Confidential: For Review Only

Table 2 – Household characteristics of children living in households with two National Child Measurement Programme participants

	Two school measurement programme-participant households (*n*=19,702)		
	n	%	95% CI[1]
**IMD quintile[2]**			
1 (most deprived)	10375	52.6	51.9,53.3
2	7836	39.8	39.1,40.5
3	1292	6.6	6.2,6.9
4	156	0.8	0.7,0.9
5 (least deprived)	43	0.2	0.2,0.3
**Number of children in the household**			
2	6449	32.8	32.1,33.4
3	7228	36.6	36.0,37.3
4 or more	6025	30.6	30.0,31.2
**Household composition**			
Working adults with children	14976	76.0	75.4,76.6
Single working age adult with children	2873	14.6	14.1,15.1
Three generation and skipped generation	1853	9.4	9.0,9.8
**Property classification**			
Flat	10260	52.1	51.4,52.8
Terraced house	8154	41.4	40.7,42.1
Other	1288	6.5	6.2,6.9

[1] Confidence interval. [2] 2015 Index of Multiple Deprivation (IMD) quintile assigned based on the child's home address postcode as recorded by the school where the child participated in the National Child Measurement Programme. The 2015 IMD accounts for socioeconomic characteristics in lower layer super output areas (LSOAs), small geographies typically comprising an average population of 1,500 people or 650 households. IMD score is derived from Indices of Deprivation, which cover seven domains: income; employment; education, skills and training; health; crime; barriers to housing and services; and living environment. The IMD score for each LSOA in England is ranked, from most to least deprived, and divided into ten equal groups indicating the most deprived 10% of LSOAs to the least deprived 10% of LSOAs, nationally. The school measurement programme dataset includes each child's IMD 2015 score and decile.

Confidential: For Review Only

Table 3 – Sociodemographic characteristics of index children living in households with two National Child Measurement Programme participants, by index child's weight status[1]

	Underweight & Healthy weight (*n*=15,275)			Overweight (*n*=2,372)			Obese (*n*=2,055)		
	n	%	95% CI[2]	n	%	95% CI[2]	n	%	95% CI[2]
**Sex**									
Male	7636	50.0	49.3,50.8	1256	52.9	50.9,54.9	1187	57.8	55.6,59.8
Female	7639	50.0	49.2,50.7	1116	47.1	45.1,49.1	868	42.2	40.2,44.4
**School year[3]**									
Reception	11219	73.4	72.7,74.1	1399	59.0	57.0,61.0	1081	52.6	50.3,54.6
Year 6	4056	26.6	25.9,27.3	973	41.0	39.0,43.0	974	47.4	45.4,49.7
**Academic year[4]**									
2013/14 & 2014/15	421	2.8	2.5,3.0	49	2.1	1.6,2.7	47	2.2	1.7,3.0
2015/16	1506	9.9	9.4,10.3	219	9.3	8.2,10.5	201	9.8	8.6,11.2
2016/17	2938	19.2	18.6,19.9	434	18.3	16.8,19.9	379	18.3	16.7,20.0
2017/18	4639	30.4	29.7,31.1	741	31.1	29.3,33.0	600	29.2	27.2,31.1
2018/19	5771	37.7	37.0,38.5	929	39.2	37.3,41.2	828	40.5	38.3,42.6
**Local authority[5]**									
City & Hackney	4001	26.2	25.5,26.9	556	23.2	21.5,24.9	441	21.5	19.8,23.3
Newham	4905	32.2	31.4,32.9	822	34.6	32.7,36.5	745	36.3	34.3,38.5
Tower Hamlets	2572	16.8	16.2,17.4	468	19.9	18.4,21.6	455	22.1	20.3,23.9
Waltham Forest	3797	24.8	24.2,25.5	526	22.3	20.7,24.0	414	20.1	18.4,21.9
**Ethnic background[6]**									
White	3739	24.5	23.8,25.2	522	22.0	20.4,23.7	354	17.2	15.6,18.9
Mixed and Other	3052	20.0	19.3,20.6	412	17.4	15.8,18.9	359	17.5	15.9,19.2
South Asian	4677	30.6	29.9,31.3	1082	45.6	43.8,47.8	1053	51.2	49.1,53.4
Black	3807	24.9	24.3,25.6	356	15.0	13.5,16.4	289	14.1	12.6,15.6

[1] National Child Measurement Programme (NCMP)-recorded body mass index (BMI) after application of ethnic-specific BMI adjustments, categorised according to UK1990 clinical reference standard: "underweight or healthy weight" (<91st centile), "overweight" (≥91st to <98th centile), or "obese" (≥98th centile). [2] Confidence interval. [3] School year of participation in the NCMP, Reception participants are aged 4-5 years and Year 6 participants are aged 10-11 years. [4] Academic year of participation in the NCMP. Academic years run from September to July. The 2013/14 and 2014/15 academic years were combined to minimise the risk of disclosing individuals. [5] Local authority of school where child participated in the NCMP. [6] As recorded in the NCMP and, where missing, supplemented with ethnic background as recorded in the child's primary care electronic health record.

Confidential: For Review Only

Figure titles and footnotes

Figure 1 – Identifying a valid Residential Anonymised Linkage Field (RALF) at the time of National Child Measurement Programme (NCMP) measurement

Children living in non-residential dwellings or where the UPRN match qualifier was not a "best" residential match were excluded (n=3,903). The match qualifier indicates how close to the place of residence the assigned UPRN is. We excluded 3,903 without residential Residential Anonymised Linkage Field (RALF) or best match RALF qualifier. In most cases (66.8%), the RALF assigned to these GP-recorded addresses was associated with a property shell, rather than the exact individual property. Others were living in sheltered accommodation or houses of multiple occupancy not further divided to enable household identification. The prevalence of overweight and obesity among the excluded children was similar to estimates among index children.

Figure 2 – Oldest child's weight status stratified by index child's weight status[1]

[1] National Child Measurement Programme (NCMP)-recorded body mass index (BMI) after application of ethnic-specific BMI adjustments, categorised according to UK1990 clinical reference standard: "underweight or healthy weight" (<91st centile), "overweight" (≥91st to <98th centile), or "obese" (≥98th centile).

Figure 3 – Multivariable[1] odds ratio estimating the likelihood of obesity[2] among the youngest children living in households with two National Child Measurement Programme participants

[1] The model including all households with two National Child Measurement Programme (NCMP) participants mutually adjusted for oldest child's weight status, number of children in the household, sex, ethnic background as recorded in the NCMP and, where missing, supplemented with ethnic background as recorded in the child's primary care electronic health record, school year of participation in the NCMP (Reception participants are aged 4-5 years and Year 6 participants are aged 10-11 years) and local authority of school where child participated in the NCMP. The model which only included households where the youngest child participated in the NCMP in Reception year mutually adjusted for the oldest child's weight status, number of children in the household, sex and ethnic background. The model which only included households where the youngest child participated in the NCMP in Year 6 mutually adjusted for the oldest child's weight status, sex and ethnic background. [2] NCMP-recorded body mass index (BMI) after application of ethnic-specific BMI adjustments, categorised according to UK1990 clinical reference standard: "obese" (≥98th centile). [3] Odds ratio and confidence interval. Odds ratios are plotted on a logarithmic scale.

Confidential: For Review Only

References

1. World Health Organisation. Childhood overweight and obesity 2019 [Available from: https://www.who.int/dietphysicalactivity/childhood/en/.
2. NHS Digital. National Child Measurement Programme, England 2018/19 School Year 2019 [Available from: https://digital.nhs.uk/data-and-information/publications/statistical/national-child-measurement-programme/2018-19-school-year.
3. Whitaker KL, Jarvis MJ, Beeken RJ, Boniface D, Wardle J. Comparing maternal and paternal intergenerational transmission of obesity risk in a large population-based sample. Am J Clin Nutr. 2010;91(6):1560-7.
4. Classen TJ. Measures of the intergenerational transmission of body mass index between mothers and their children in the United States, 1981-2004. Econ Hum Biol. 2010;8(1):30-43.
5. Johnson PCD, Logue J, McConnachie A, Abu-Rmeileh NME, Hart C, Upton MN, et al. Intergenerational change and familial aggregation of body mass index. European Journal of Epidemiology. 2011;27(1):53-61.
6. Kracht CL, Sisson SB. Sibling influence on children's objectively measured physical activity: a meta-analysis and systematic review. BMJ Open Sport & Exercise Medicine. 2018;4(1):e000405.
7. Meller FO, Loret de Mola C, Assuncao MCF, Schafer AA, Dahly DL, Barros FC. Birth order and number of siblings and their association with overweight and obesity: a systematic review and meta-analysis. Nutr Rev. 2018;76(2):117-24.
8. Meller FO, Assunção MCF, Schäfer AA, De Mola CL, Barros AJD, Dahly DL, et al. The influence of birth order and number of siblings on adolescent body composition: evidence from a Brazilian birth cohort study. British Journal of Nutrition. 2015;114(1):118-25.
9. Park SH, Cormier E. Influence of Siblings on Child Health Behaviors and Obesity: A Systematic Review. Journal of Child and Family Studies. 2018;27(7):2069-81.
10. Chanfreau J, Barclay K, Keenan K, Goisis A. Sibling group size and BMI over the life course: Evidence from four British cohort studies. Advances in Life Course Research. 2022;53.
11. Mosli RH, Kaciroti N, Corwyn RF, Bradley RH, Lumeng JC. Effect of Sibling Birth on BMI Trajectory in the First 6 Years of Life. Pediatrics. 2016;137(4):e20152456.
12. Pachucki MC, Lovenheim MF, Harding M. Within-family obesity associations: evaluation of parent, child, and sibling relationships. Am J Prev Med. 2014;47(4):382-91.
13. Khoury P, Morrison JA, Laskarzewski PM, Glueck CJ. Parent-offspring and sibling body mass index associations during and after sharing of common household environments: The princeton school district family study. Metabolism. 1983;32(1):82-9.
14. Park SH, Cormier E, Grove K, Kelley SJ, Faith MS. Health-related behaviors and weight-related outcomes within sibling pairs: A systematic review. Journal of Pediatric Nursing. 2023;73:e154-e63.
15. Office for Health Improvement & Disparities. Public Health Profiles [Available from: https://fingertips.phe.org.uk Crown Copyright 2023.
16. Office for Health Improvement & Disparities. National Child Measurement Programme 2022: information for schools: Department of Health and Social Care; [Available from: https://www.gov.uk/government/publications/national-child-measurement-programme-operational-guidance/national-child-measurement-programme-2022-information-for-schools.
17. Department for Education. Schools and Pupils by Type of School, Borough: Department for Education; [Available from: https://data.london.gov.uk/dataset/schools-and-pupils-type-school-borough.
18. Discovery Data Service. Discovery Data Service [Available from: https://www.endeavourhealth.org/DDS.html.
19. NHS Digital. Validation of National Child Measurement Programme data: NHS Digital; [Available from: https://digital.nhs.uk/services/national-child-measurement-programme/it-system/validation-of-national-child-measurement-programme-data.
20. Hippisley-Cox J. OpenPseudonymiser: University Of Nottingham; [Available from: https://www.openpseudonymiser.org/.
21. NHS England. About information governance: National Health Service; [Available from: https://www.england.nhs.uk/ig/about/.
22. Harper G, Stables D, Simon P, Ahmed Z, Smith K, Robson J, et al. Evaluation of the ASSIGN open-source deterministic address-matching algorithm for allocating unique property reference numbers to general practitioner-recorded patient addresses. Int J Popul Data Sci. 2021;6(1):1674.

Confidential: For Review Only

23. Scientific Advisory Committee on Nutrition, Royal College of Paediatrics and Child Health. Consideration of issues around the use of BMI centile thresholds for defining underweight, overweight and obesity in children aged 2-8 years in the UK. 2012.
24. Hudda MT, Nightingale CM, Donin AS, Fewtrell MS, Haroun D, Lum S, et al. Body mass index adjustments to increase the validity of body fatness assessment in UK Black African and South Asian children. Int J Obes. 2017;41(7):1048-55.
25. NHS. Ethnic category code: NHS Digital; [Available from: https://www.datadictionary.nhs.uk/data_elements/ethnic_category.html?hl=ethnic%2Ccategory.
26. Department for Communities and Local Government. The English Indices of Deprivation 2015 - Frequently Asked Questions (FAQs). 2016.
27. Harper G, Mayhew L. Using Administrative Data to Count and Classify Households with Local Applications. Applied Spatial Analysis and Policy. 2015;9(4):433-62.
28. Firman N, Robson J, Ahmed Z, Boomla K, Dezateux C. Completeness and representativeness of body mass index in children's electronic general practice records: Linked cross-sectional study in an ethnically-diverse urban population in the United Kingdom. Pediatric Obesity. 2021;16(8):e12772.
29. Faith MS, Pietrobelli A, Heo M, Johnson SL, Keller KL, Heymsfield SB, et al. A twin study of self-regulatory eating in early childhood: estimates of genetic and environmental influence, and measurement considerations. International Journal of Obesity. 2012;36(7):931-7.
30. Osai KV, Dorsch TE, Whiteman SD. “To Be, or Not to Be, That Is the Question”: Modeling and Differentiation Among Siblings Participating in Organized Youth Sport. Journal of Sport & Exercise Psychology. 2020;42(6):500-10.
31. Whiteman SD, Christiansen A. Processes of Sibling Influence in Adolescence: Individual and Family Correlates*. Family Relations. 2008;57(1):24-34.
32. Gustafson SL, Rhodes RE. Parental Correlates of Physical Activity in Children and Early Adolescents. Sports Medicine. 2006;36(1):79-97.
33. Nielsen LA, Nielsen TR, Holm JC. The Impact of Familial Predisposition to Obesity and Cardiovascular Disease on Childhood Obesity. Obes Facts. 2015;8(5):319-28.
34. Office for National Statistics. Families and households in the UK: 2019 [Available from: https://www.ons.gov.uk/peoplepopulationandcommunity/birthsdeathsandmarriages/families/bulletins/familiesandhouseholds/2019#multi-family-households-are-the-fastest-growing-household-type-in-the-uk-but-currently-represent-the-smallest-share-of-households.

Confidential: For Review Only

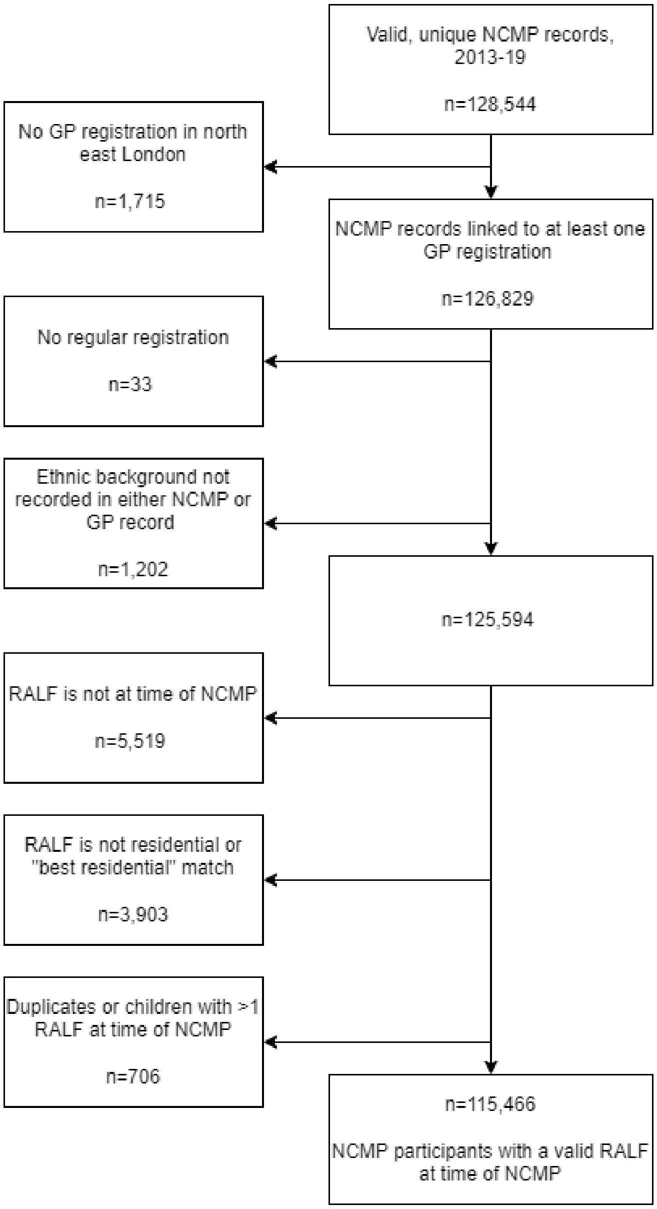

Identifying a valid Residential Anonymised Linkage Field (RALF) at the time of National Child Measurement Programme (NCMP) measurement

Children living in non-residential dwellings or where the UPRN match qualifier was not a “best” residential match were excluded (n=3,903). The match qualifier indicates how close to the place of residence the assigned UPRN is.

141x261mm (72 x 72 DPI)

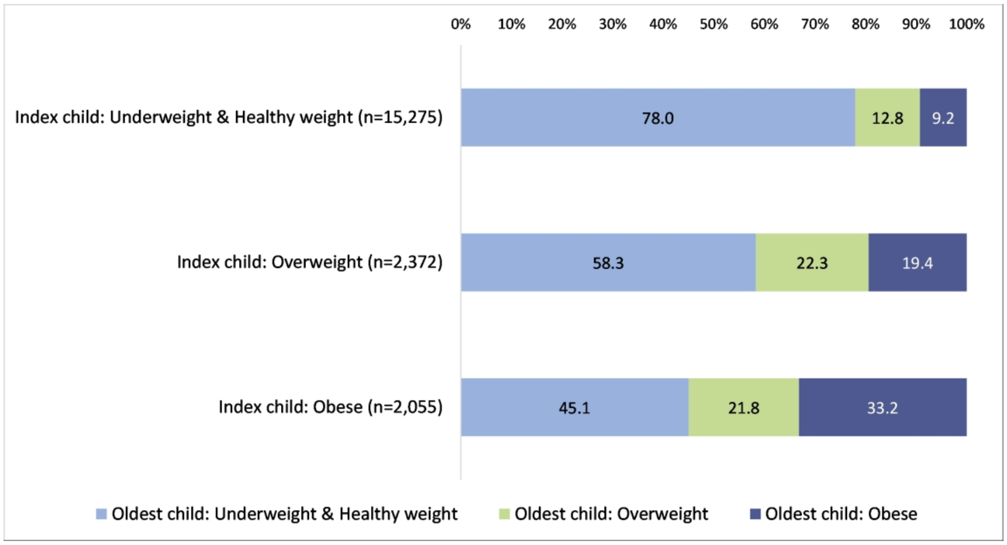

Oldest child's weight status stratified by index child's weight status[1]

[1] National Child Measurement Programme (NCMP)-recorded body mass index (BMI) after application of ethnic-specific BMI adjustments, categorised according to UK1990 clinical reference standard: "underweight or healthy weight" (<91st centile), "overweight" (≥91st to <98th centile), or "obese" (≥98th centile).

554x298mm (118 x 118 DPI)

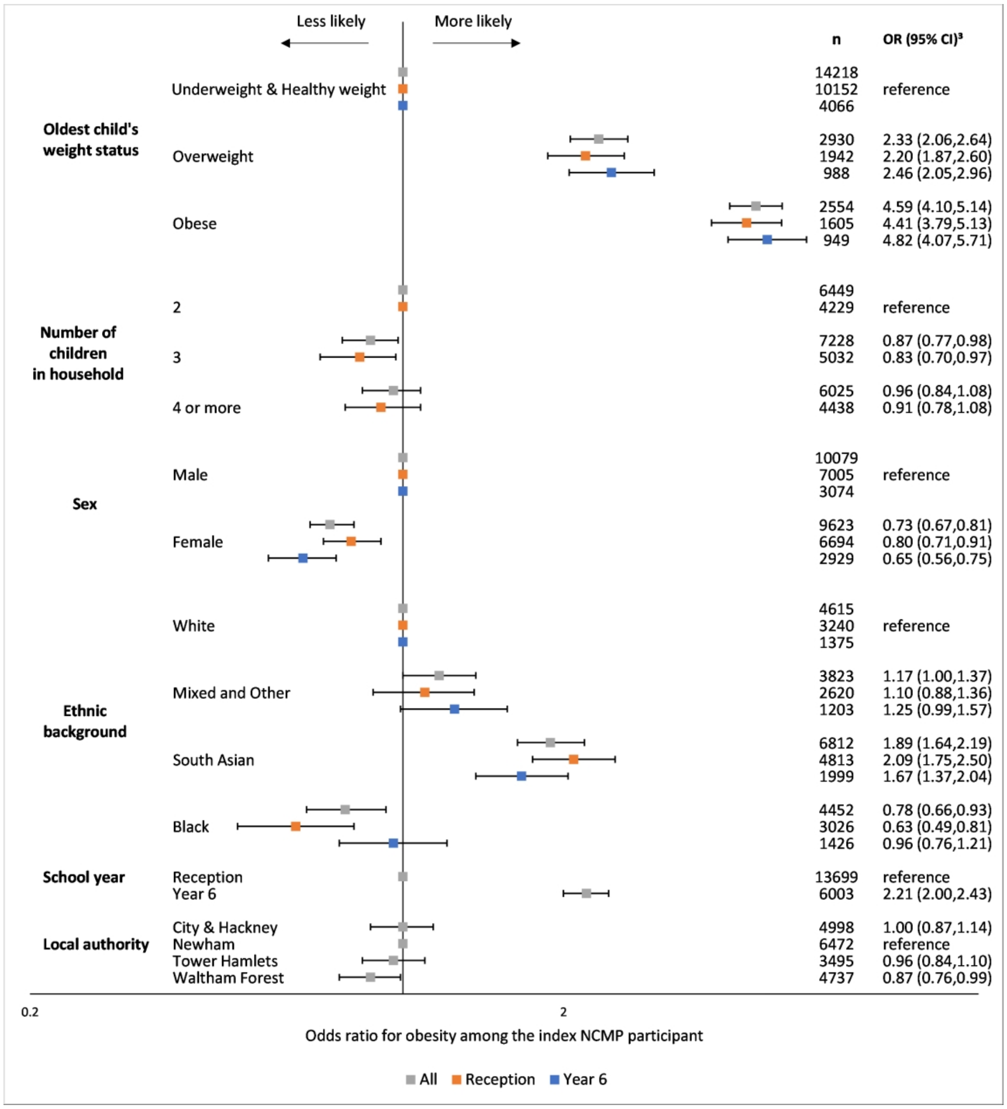

Multivariable[1] odds ratio estimating the likelihood of obesity[2] among the youngest children living in households with two National Child Measurement Programme participants

[1] The model including all households with two National Child Measurement Programme (NCMP) participants mutually adjusted for oldest child's weight status, number of children in the household, sex, ethnic background as recorded in the NCMP and, where missing, supplemented with ethnic background as recorded in the child's primary care electronic health record, school year of participation in the NCMP (Reception participants are aged 4-5 years and Year 6 participants are aged 10-11 years) and local authority of school where child participated in the NCMP. The model which only included households where the youngest child participated in the NCMP in Reception year mutually adjusted for the oldest child's weight status, number of children in the household, sex and ethnic background. The model which only included households where the youngest child participated in the NCMP in Year 6 mutually adjusted for the oldest child's weight status, sex and ethnic background. [2] NCMP-recorded body mass index (BMI) after application of ethnic-specific BMI adjustments, categorised according to UK1990 clinical reference standard: "obese" (≥98th centile). [3] Odds ratio and confidence interval. Odds ratios are plotted on a logarithmic scale.

577x635mm (118 x 118 DPI)

Is obesity more likely among children sharing a household with an older child with obesity? Cross-sectional study of linked National Child Measurement Programme data and electronic health records

Nicola Firman,[1] Marta Wilk,[1] Milena Marszalek,[1] Lucy Griffiths,[2] Gill Harper,[1] Carol Dezateux[1]

[1] Centre for Primary Care, Wolfson Institute of Population Health, Faculty of Medicine and Dentistry, Queen Mary University of London, Yvonne Carter Building, 58 Turner Street, London, E1 2AB

[2] Swansea University Medical School, Faculty of Medicine, Health & Life Sciences

Supplementary material

Figure S1 – Relationship between address and General Practice (GP) registration dates and Residential Anonymised Linkage Fields (RALFs) at the time of National Child Measurement Programme (NCMP)

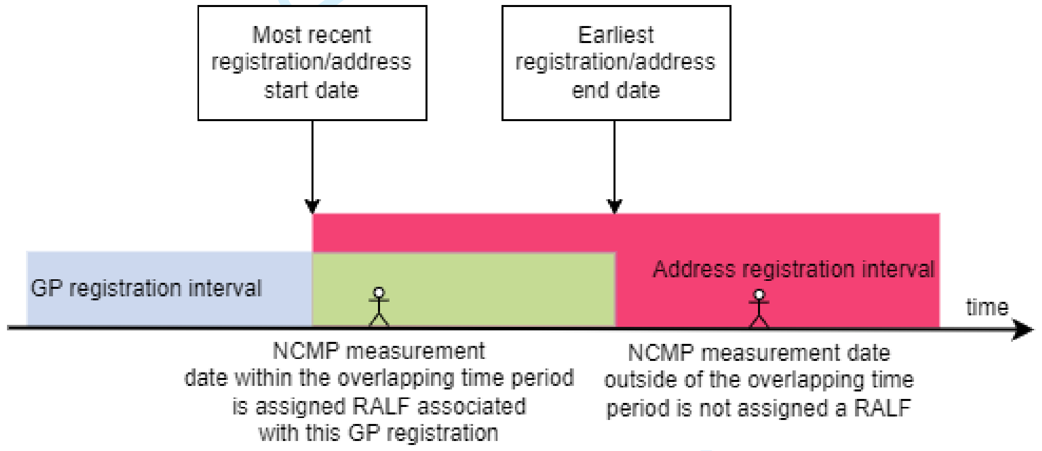

For peer review only

Figure S2 – Identifying National Child Measurement Programme (NCMP) participants' valid General Practice (GP)-recorded Residential Anonymised Linkage Fields (RALFs)

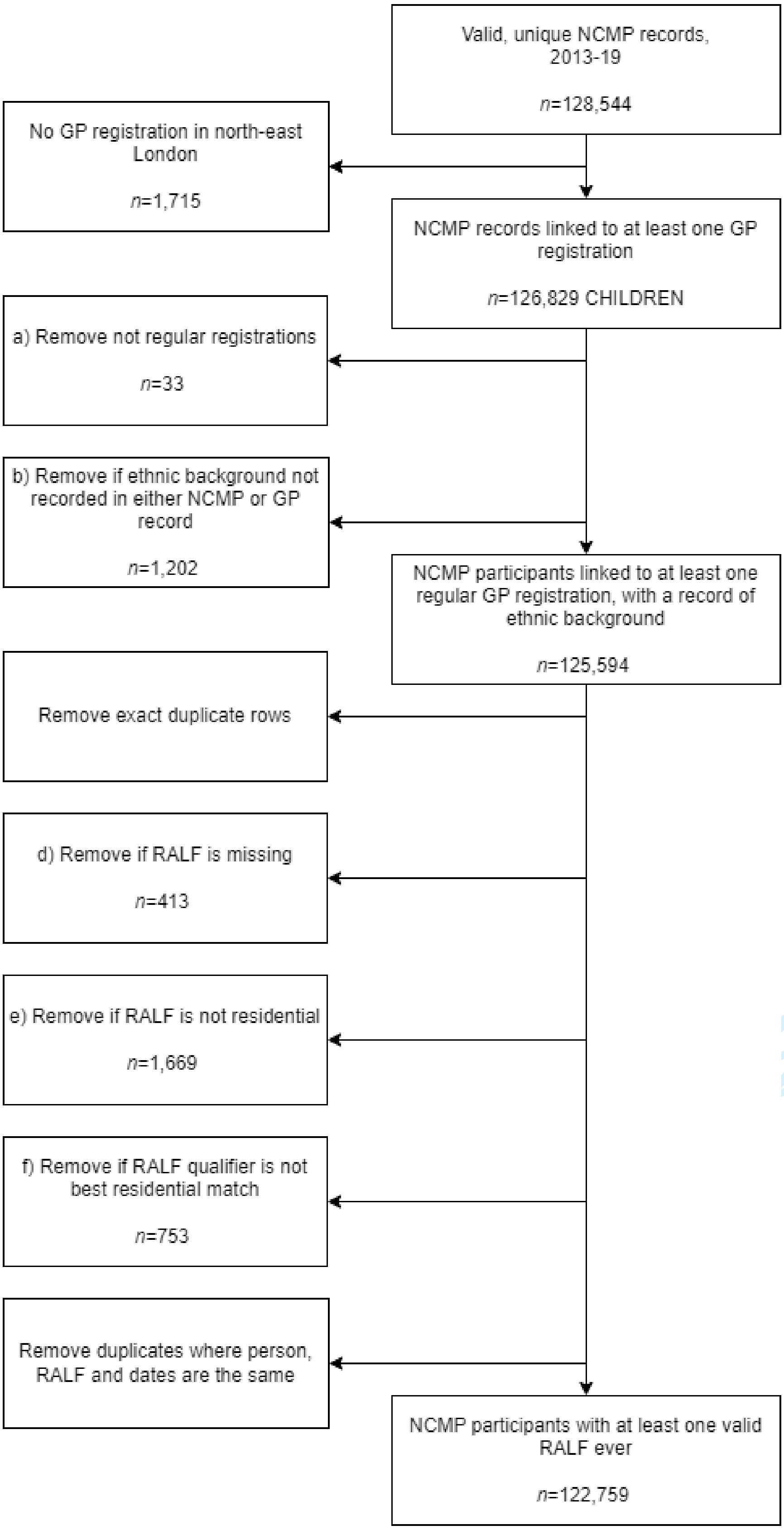

For peer review only

Figure S3 – Identifying National Child Measurement Programme (NCMP) participants sharing a household using Residential Anonymised Linkage Fields (RALFs)

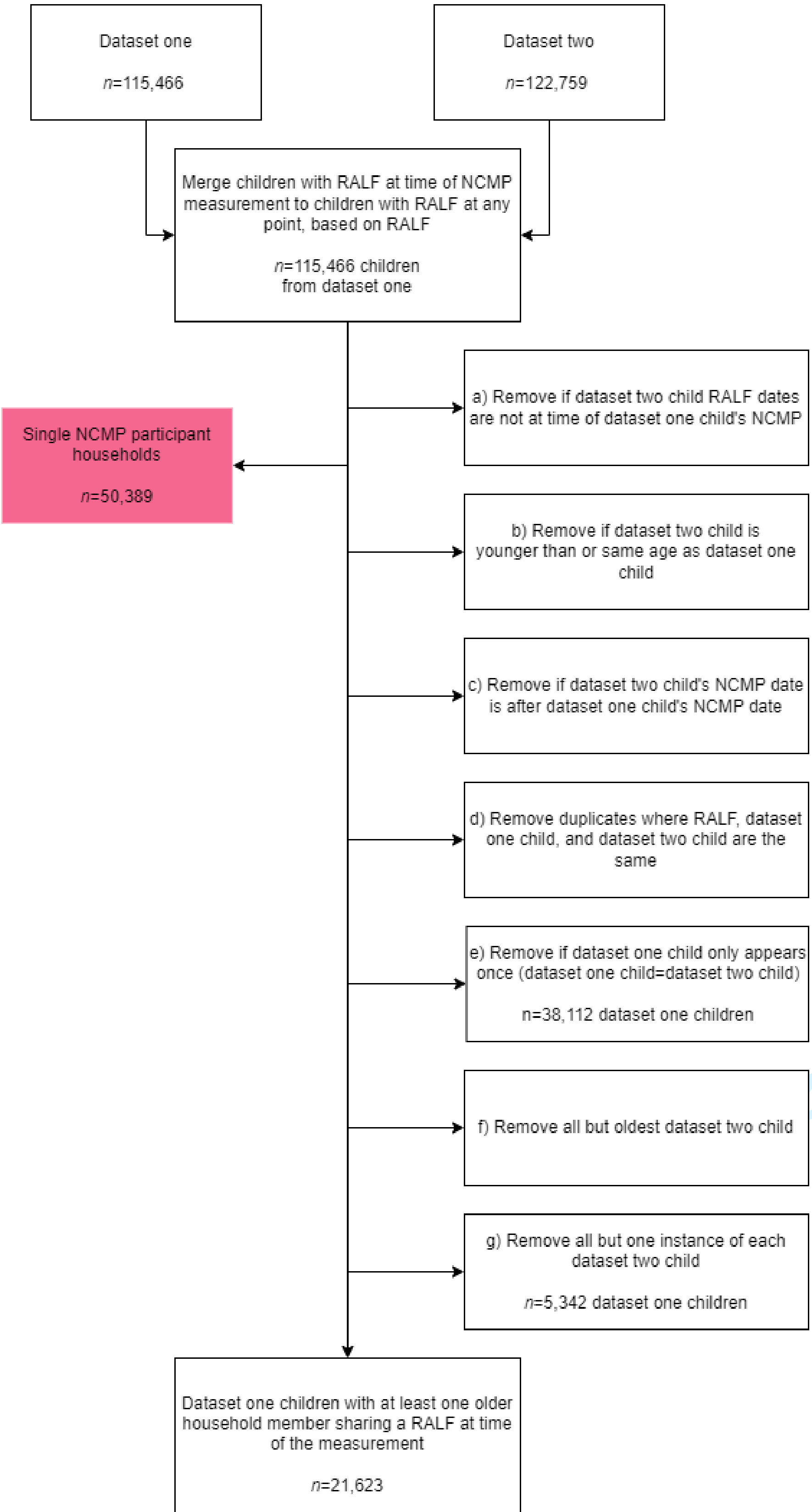

Index children were joined with children in dataset two using Stata's many to many `joinby` *function, combining all pairwise combinations on common RALFs. Where an older child matched to multiple index children, only one random pair was retained (n=5,342).*

Only

Figure S4 – Identifying all other household members at the time of the index child's NCMP measurement

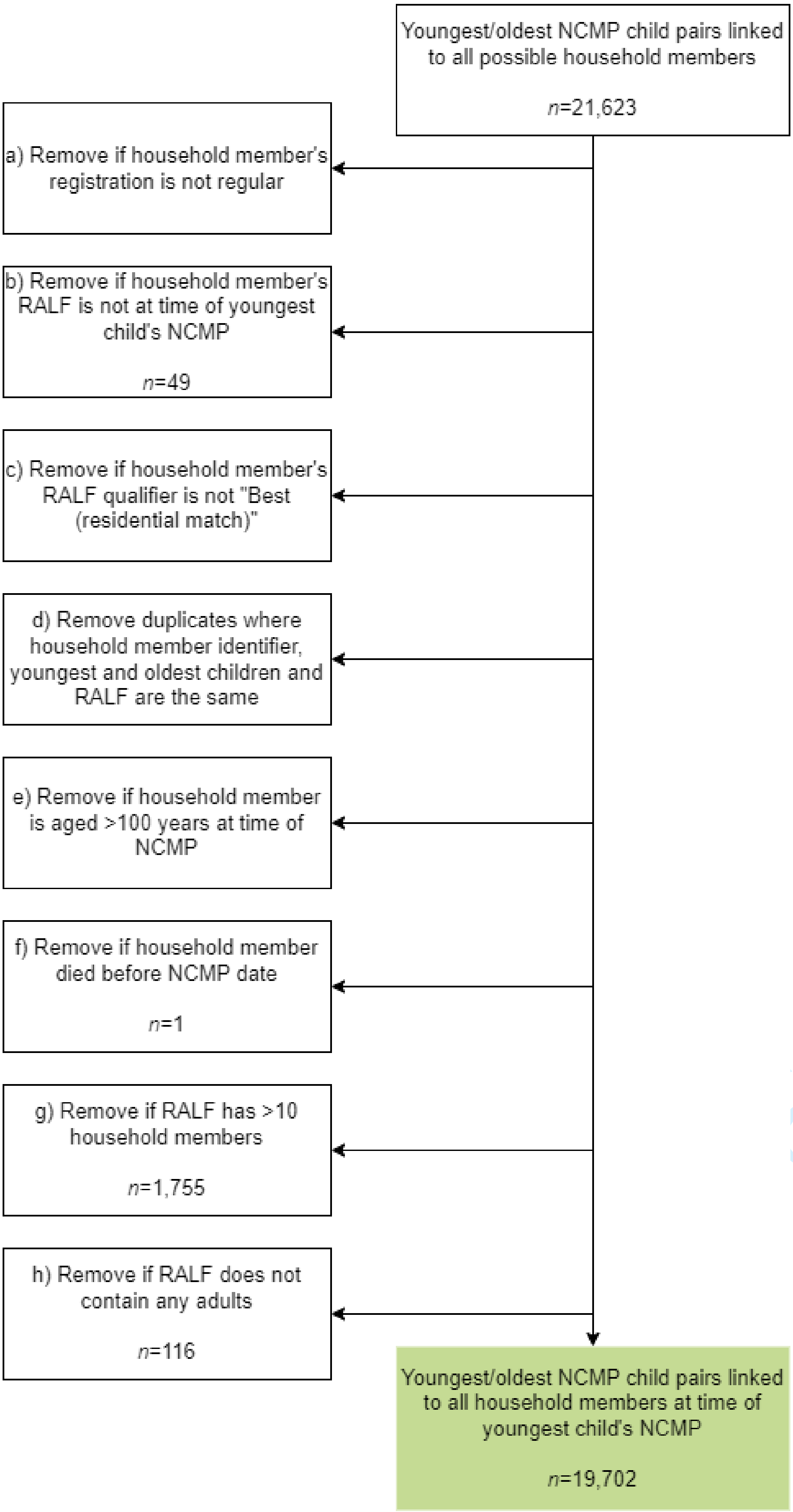

For each household member, age at the time of the youngest child's NCMP measurement was calculated.

Table S1 – Sample characteristics of oldest National Child Measurement Programme participants in the household

The distribution by sex, ethnic background and weight status was broadly representative of NCMP participants in the four local authorities as a whole.

	**All (*n*=19,702)**			**Reception (*n*=6,506)**			**Year 6 (*n*=13,196)**		
	**n**	**%**	**95% CI[1]**	**n**	**%**	**95% CI[1]**	**n**	**%**	**95% CI[1]**
**Sex**									
Male	9975	50.6	49.9,51.3	3268	50.1	48.9,51.3	6707	50.9	50.0,51.7
Female	9727	49.4	48.7,50.1	3238	49.9	48.7,51.1	6489	49.1	48.3,50.0
**School year[2]**									
Reception	6506	33.0	32.3,33.6	6506	100.0				
Year 6	13196	67.0	66.4,67.7				13196	100.0	
**Academic year[3]**									
2013/14	3387	17.2	16.7,17.7	1418	21.8	20.8,22.9	1969	14.9	14.3,15.5
2014/15	3820	19.4	18.9,20.0	1412	21.7	20.7,22.7	2408	18.3	17.6,19.0
2015/16	5888	29.9	29.3,30.5	2075	31.9	30.8,33.0	3813	28.9	28.1,29.7
2016/17	1435	21.0	20.4,21.6	1251	19.2	18.3,20.2	2884	21.9	21.2,22.6
2017/18 & 2018/19	2472	12.5	12.1,13.0	350	5.3	4.9,6.0	2122	16.0	15.5,16.7
**Local authority[4]**									
City & Hackney	5041	25.6	25.0,26.2	1848	28.4	27.4,29.5	3193	24.2	23.5,24.9
Newham	3460	17.5	17.0,18.1	864	13.3	12.5,14.1	2596	19.6	19.0,20.3
Tower Hamlets	6458	32.8	32.1,33.5	2006	30.8	29.6,31.9	4452	33.8	33.0,34.6
Waltham Forest	4743	24.1	23.5,24.7	1788	27.5	26.5,28.6	2955	22.4	21.7,23.1
**Ethnic background[5]**									
White	4503	22.9	22.3,23.4	1873	28.8	27.7,29.9	2630	19.9	19.3,20.6
Mixed and Other	4118	20.9	20.3,21.5	1361	20.9	20.0,21.9	2757	20.9	20.2,21.6
South Asian	6857	34.8	34.1,35.5	1917	29.5	28.4,30.6	4940	37.4	36.6,38.3
Black	4224	21.4	20.9,22.0	1355	20.8	19.8,21.8	2869	21.7	21.1,22.5
**Weight status[6]**									
Underweight	280	1.4	1.3,1.6	97	1.5	1.2,1.8	183	1.4	1.2,1.6
Healthy weight	13938	70.7	70.1,71.4	5299	81.4	80.5,82.4	8639	65.5	64.7,66.3
Overweight	2930	14.9	14.4,15.4	679	10.4	9.7,11.2	2251	17.0	16.4,17.7
Obese	2554	13.0	12.5,13.4	431	6.7	6.1,7.3	2123	16.1	15.5,16.7

[1] Confidence interval. [2] School year of participation in the National Child Measurement Programme (NCMP), Reception participants are aged 4-5 years and Year 6 participants are aged 10-11 years. [3] Academic year of participation in the NCMP. Academic years run from September to July. The 2017/18 and 2018/19 academic years were combined to minimise risk of disclosure of individuals. [4] Local authority of school where child participated in the NCMP. [5] As recorded in the NCMP and, where missing, supplemented with ethnic background as recorded in the child's primary care electronic health record. [6] NCMP-recorded body mass index (BMI) after application of ethnic-specific BMI adjustments, categorised according to UK1990 clinical reference standard: "underweight" (BMI<2nd centile), "healthy weight" (≥2nd to <91st centile), "overweight" (≥91st to <98th centile), or "obese" (≥98th centile).

Confidential: For Review Only

Table S2 – Weight status distribution of index children participating in the National Child Measurement Programme, using UK1990 clinical and International Obesity Task Force cut-offs

	All (*n*=19,702)			Reception (*n*=13,699)			Year 6 (*n*=6,003)		
	n	%	95% CI[1]	n	%	95% CI[1]	n	%	95% CI[1]
**UK1990 clinical reference standard**									
Underweight	270	1.4	1.2,1.5	194	1.4	1.2,1.6	76	1.3	1.0,1.6
Healthy weight	15005	76.2	75.6,76.8	11025	80.5	79.9,81.2	3980	66.3	65.1,67.5
Overweight	2372	12.0	11.5,12.4	1399	10.2	9.7,10.7	973	16.1	15.2,17.0
Obese	2055	10.4	10.0,10.9	1081	7.9	7.4,8.3	974	16.3	15.4,17.3
**International Obesity Task Force cut-offs**									
Underweight	1409	7.1	6.8,7.5	1037	7.6	7.1,8.0	372	6.2	5.6,6.8
Healthy weight	13339	67.7	67.0,68.4	9682	70.7	69.9,71.4	3657	60.9	59.7,62.1
Overweight	3485	17.7	17.2,18.2	2054	15.0	14.4,15.6	1431	23.8	22.8,24.9
Obese	1469	7.5	7.1,7.8	926	6.7	6.4,7.2	543	9.1	8.3,9.8

[1] *Confidence interval.*

Table S3 – Household characteristics of children living in households with two National Child Measurement Programme participants, by index child's weight status[1]

	Youngest child (*n*=19 702)								
	Underweight & Healthy weight (*n*=15 275)			Overweight (*n*=2 372)			Obese (*n*=2 055)		
**Number of children in the household**									
2	4 992	32.7	31.9,33.4	789	33.3	31.6,35.4	668	32.5	30.5,34.5
3	5 596	36.6	35.8,37.4	899	37.9	35.9,39.8	733	35.7	33.7,37.8
4 or more	4 687	30.7	30.0,31.5	684	28.8	26.9,30.6	654	31.8	29.8,33.8
**Household composition**									
Adults with children	11 593	75.9	75.2,76.6	1 797	75.8	74.0,77.5	1 586	77.2	75.3,78.9
Single adult with children	2 282	14.9	14.4,15.5	333	14.0	12.7,15.5	258	12.5	11.2,14.0
Three generation and skipped generation	1 400	9.2	8.7,9.6	242	10.2	9.0,11.5	211	10.3	9.1,11.7
**Property classification**									
Flat	7 887	51.6	50.8,52.4	1 253	52.8	50.8,54.8	1 120	54.5	52.3,56.6
Terraced house	6 375	41.7	41.0,42.5	970	40.9	38.9,42.9	809	39.4	37.3,41.5
Other	1 013	6.6	6.2,7.0	149	6.3	5.4,7.3	126	6.1	5.2,7.3

[1] *National Child Measurement Programme (NCMP)-recorded body mass index (BMI) after application of ethnic-specific BMI adjustments, categorised according to UK1990 clinical reference standard: "underweight or healthy weight" (<91st centile), "overweight" (≥91st to <98th centile), or "obese" (≥98th centile).* [2] *Confidence interval.*

Confidential: For Review Only

Table S4 – Characteristics of index and oldest child pairs, by index child's weight status[1]

	**Underweight & Healthy weight (*n*=15 275)**			**Overweight (*n*=2 372)**			**Obese (*n*=2 055)**		
	**n**	**%**	**95% CI[2]**	**n**	**%**	**95% CI[2]**	**n**	**%**	**95% CI[2]**
**Oldest child's weight status[1]**									
Underweight	256	1.7	1.5,1.9	13	0.5	0.3,0.9	11	0.5	0.3,1.0
Healthy weight	11 652	76.3	75.6,76.9	1 371	57.8	55.7,59.7	915	44.5	42.5,46.8
Overweight	1 955	12.8	12.3,13.3	528	22.3	20.8,24.1	447	21.8	19.9,23.5
Obese	1 412	9.2	8.8,9.7	460	19.4	17.8,21.0	682	33.2	31.2,35.2
**Sex concordance[3]**									
Different	7 681	50.0	49.4,51.0	1 174	49.5	47.5,51.5	1 049	51.0	48.8,53.1
Same	7 594	49.7	49.0,50.6	1 198	50.5	48.5,52.5	1 006	49.0	46.9,51.2
**Time difference (months)[4]**									
Median (IQR[5])	23.3	12.6,33.1		23.4	12.6,33.0		22.9	12.5,31.9	

[1] National Child Measurement Programme (NCMP)-recorded body mass index (BMI) after application of ethnic-specific BMI adjustments, categorised according to UK1990 clinical reference standard: "underweight" (BMI<2nd centile), "healthy weight" (≥2nd to <91st centile), "overweight" (≥91st to <98th centile), or "obese" (≥98th centile). [2] Confidence interval. [3] Whether the youngest and oldest child were of the same or different sex. [4] Time difference, calculated in months, between the oldest and youngest child's NCMP measurements. [5] Interquartile range.

Confidential: For Review Only

Table S5 – Univariable and multivariable odds ratios estimating the likelihood of obesity[1] among index children living in households with two National Child Measurement Programme participants

	All (*n*=19 702)				Reception (*n*=13 699)				Year 6 (*n*=6 003)			
	Univariable		Multivariable[2]		Univariable		Multivariable[3]		Univariable		Multivariable	
	OR[5]	95% CI[6]	OR[5]	95% CI[6]	OR[5]	95% CI[6]	OR[5]	95% CI[6]	OR[5]	95% CI[6]	OR[5]	95% CI[6]
**Oldest child's weight status[7]**												
Underweight & Healthy weight (reference)	1		1		1		1		1		1	
Overweight	2.58	2.29,2.92	2.33	2.06,2.64	2.46	2.09,2.90	2.20	1.87,2.60	2.57	2.14,3.08	2.46	2.05,2.96
Obese	5.23	4.68,5.84	4.59	4.10,5.14	5.00	4.31,5.80	4.41	3.79,5.13	5.05	4.27,5.97	4.82	4.07,5.71
**Number of children in the household**												
2 (reference)	1		1		1		1		1			
3	0.98	0.87,1.09	0.87	0.77,0.98	1.00	0.86,1.17	0.83	0.70,0.97	1.03	0.87,1.21		
4 or more	1.05	0.94,1.18	0.96	0.84,1.08	1.13	0.97,1.32	0.91	0.78,1.08	1.14	0.96,1.35		
**Household composition**												
Adults with children (reference)	1				1				1			
Single adult with children	0.83	0.73,0.96			0.75	0.61,0.91			0.91	0.74,1.40		
Three and skipped generation	1.08	0.93,1.26			1.04	0.85,1.29			1.12	0.89,1.40		
**Sex**												
Male (reference)	1		1		1		1		1		1	
Female	0.74	0.68,0.81	0.73	0.67,0.81	0.81	0.71,0.92	0.80	0.71,0.91	0.66	0.58,0.76	0.65	0.56,0.75
**Ethnic background[8]**												
White (reference)	1		1		1		1		1		1	
Mixed and Other	1.25	1.07,1.45	1.17	1.00,1.37	1.13	0.91,1.40	1.10	0.88,1.36	1.36	1.08,1.70	1.25	0.99,1.57
South Asian	2.20	1.94,2.49	1.89	1.64,2.19	2.40	2.02,2.84	2.09	1.75,2.50	2.02	1.67,2.46	1.67	1.37,2.04
Black	0.84	0.71,0.98	0.78	0.66,0.93	0.61	0.48,0.78	0.63	0.49,0.81	1.04	0.83,1.31	0.96	0.76,1.21
**School year[9]**												
Reception (reference)	1		1									
Year 6	2.26	2.06,2.48	2.21	2.00,2.43								

Confidential: For Review Only

	All (*n*=19 702)				Reception (*n*=13 699)				Year 6 (*n*=6 003)			
	Univariable		Multivariable[2]		Univariable		Multivariable[3]		Univariable		Multivariable	
	OR[5]	95% CI[6]	OR[5]	95% CI[6]	OR[5]	95% CI[6]	OR[5]	95% CI[6]	OR[5]	95% CI[6]	OR[5]	95% CI[6]
**Academic year[10]**												
2013/14	0.70	0.32,1.52			0.86	0.37,1.99						
2014/15	0.83	0.60,1.16			1.01	0.69,1.49			1.17	0.58,2.34		
2015/16	0.94	0.80,1.11			1.16	0.95,1.43			0.93	0.70,1.24		
2016/17	0.91	0.80,1.03			1.01	0.85,1.21			0.94	0.78,1.15		
2017/18	0.90	0.81,1.01			0.93	0.79,1.08			0.94	0.80,1.11		
2018/19 (reference)	1				1				1			
**Local authority[11]**												
City & Hackney	0.74	0.66,0.84	1.00	0.87,1.14	0.67	0.57,0.80			0.84	0.70,1.01		
Newham (reference.)	1		1		1				1			
Tower Hamlets	1.15	1.02,1.30	0.96	0.84,1.10	1.25	1.06,1.47			1.13	0.92,1.38		
Waltham Forest	0.74	0.65,0.84	0.87	0.76,0.99	0.69	0.58,0.83			0.76	0.63,0.92		
**Sex concordance[12]**												
Different (reference.)	1				1				1			
Same	0.97	0.88,1.06			0.96	0.85,1.08			1.00	0.87,1.14		
**Time difference (months)[13]**												
	1.00	0.99,1.00			0.99	0.99,1.00			1.00	0.99,1.00		

[1] National Child Measurement Programme (NCMP)-recorded body mass index (BMI) after application of ethnic-specific BMI adjustments, categorised according to UK1990 clinical reference standard: "obese" (≥98th centile). [2] Mutually adjusting for oldest child's weight status, number of children in the household, sex, ethnic background and school year. [3] Mutually adjusting for the oldest child's weight status, number of children in the household, sex and ethnic background. [4] Mutually adjusting for the oldest child's weight status, sex and ethnic background. [5] Odds ratio. [6] Confidence interval. [7] National Child Measurement Programme (NCMP)-recorded body mass index (BMI) after application of ethnic-specific BMI adjustments, categorised according to UK1990 clinical reference standard: "underweight or healthy weight" (<91st centile), "overweight" (≥91st to <98th centile), or "obese" (≥98th centile). [8] As recorded in the NCMP and, where missing, supplemented with ethnic background as recorded in the child's primary care electronic health record. [9] School year of participation in the National Child Measurement Programme (NCMP), Reception participants are aged 4-5 years and Year 6 participants are aged 10-11 years. [10] Academic year of participation in the National Child Measurement Programme (NCMP). Academic years run from September to July. [11] Local authority of school where child participated in the NCMP. [12] Whether the youngest and oldest child were of the same or different sex. [13] Time difference, calculated in months, between the oldest and youngest child's NCMP measurements. Cells highlighted in bold indicate occasions where the estimates are significantly difference to the reference group in the multivariable models.

Confidential: For Review Only
